# Hypertension promotes neuroinflammation, brain injury and cognitive impairment

**DOI:** 10.1016/j.bbih.2025.101059

**Published:** 2025-07-10

**Authors:** Quynh Nhu Dinh, Antony Vinh, Cecilia Lo, David E. Wong Zhang, Hericka Bruna Figueiredo Galvao, Sharmelee Selvaraji, Hyun Ah Kim, Sophocles Chrissobolis, Thiruma V. Arumugam, Grant R. Drummond, Christopher G. Sobey, T. Michael De Silva

**Affiliations:** a Centre for Cardiovascular Biology and Disease Research and La Trobe Institute for Molecular Sciences (LIMS), La Trobe University, Victoria, Australia; b Department of Microbiology, Anatomy, Physiology & Pharmacology, School of Agriculture, Biomedicine, Environment, La Trobe University, Victoria, Australia; c Memory Aging and Cognition Centre, Department of Pharmacology, National University of Singapore, Singapore; d Department of Pharmaceutical and Biomedical Sciences, Raabe College of Pharmacy, Ohio Northern University, Ohio, USA

**Keywords:** Hypertension, Inflammation, Brain, Cognition, Blood-brain barrier

## Abstract

**Background:**

Hypertension increases the risk for cognitive impairment and promotes vascular and renal inflammation. We tested if immune cell infiltration occurs in the brain during hypertension and if it is associated with cognitive impairment.

**Methods:**

Male C57Bl/6 mice were administered vehicle, angiotensin II (0.7 mg/kg/d S.C.) or aldosterone (0.72 mg/kg/d S.C.) via osmotic minipumps. A subset of mice also received hydralazine (50 mg/kg) in their drinking water after minipump implantation. We measured systolic blood pressure, markers of inflammation, working memory and transcriptomic changes in the brain.

**Results:**

Administration of angiotensin II or aldosterone increased blood pressure and promoted blood-brain barrier dysfunction, leukocyte accumulation and impairment of working memory in mice. When co-administered with angiotensin II, hydralazine prevented the development of these changes. In a separate cohort of mice in which angiotensin II-induced changes were first established, intervention with hydralazine lowered blood pressure but did not reverse brain inflammation or cognitive impairment. Finally, angiotensin II infusion altered the transcriptomic profile of the whole brain, as well as specifically within the hippocampus, and co-treatment with hydralazine modulated these changes.

**Conclusions:**

Experimental hypertension leads to brain inflammation and was associated with impaired working memory. Cognitive impairment that develops during hypertension can be inhibited, but not readily reversed, by anti-hypertensive therapy.

## Highlights

• Lowering blood pressure can prevent but not reverse cognitive impairment during hypertension.
• The cognitive impairment is associated with neuroinflammation.
• Infusion with angiotensin II alters the transcriptome in the whole brain and specifically in the hippocampus.
• Neuroinflammation, IgG deposition, and cognitive impairmentare dependent on the degree of elevated blood pressure.

## Abbreviations

CCL -: CC motif ligand
CCR –: CC motif receptor
CD –: Cluster of differentiation
Cldn5 -: Claudin-5
DESeq -: Differential expression analysis for sequence
FKPM -: Fragments per kilobase of transcript per million mapped reads
GO -: Gene ontology
GSEA -: Gene set enrichment analysis
IL –: Interleukin
IgG: immunoglobulin G
mRNA: messenger RNA
ORA –: Overrepresentation analysis
PBS: phosphate-buffered saline
SPRINT -: Systolic Blood Pressure Intervention Trial
SPRINT MIND -: Systolic Blood Pressure Intervention Trial Memory and Cognition in Decreased Hypertension
Tjp1 –: Tight junction protein 1
ZO-1 -: Zonula occludens 1

## 1 Introduction

Hypertension is a prevalent condition affecting at least 30 % of the global adult population (Mills et al., 2016). The impact of uncontrolled hypertension is particularly pronounced in the brain and cerebral circulation, where end-organ disease manifests earlier than in other parts of the body (Jennings and Zanstra, 2009). Hypertension is a major risk factor for two of the most important conditions that impact the brain: stroke and cognitive impairment (Iadecola et al., 2016). However, the underlying mechanisms by which hypertension promotes brain disease are not clear. Importantly, the incidence of cognitive impairment is rapidly increasing and even mild cognitive decline increases the risk of developing dementia (Bozoki et al., 2001). Patients on anti-hypertensives have a lower risk of cognitive impairment and dementia (Ou et al., 2020) but it is not conclusive from clinical trials whether anti-hypertensive therapy reverses established cognitive dysfunction (Iadecola et al., 2016; Rapp et al., 2020).

Inflammation plays a central role in the pathophysiology of hypertension (Drummond et al., 2019). For example, mice that lack T cells have reduced pressor responses (Guzik et al., 2007; Dinh et al., 2021) and preventing T cell activation attenuates hypertension (Dinh et al., 2021; Vinh et al., 2010). Hypertension is strongly associated with inflammation and leukocyte infiltration into the systemic vasculature and kidneys. While T cells and microglia are upregulated in the brain during hypertension, it remains unclear whether other leukocyte subtypes are similarly upregulated. Angiotensin II infusion has been shown to stimulate tumour necrosis factor (TNF)-α production in the hippocampus (Iulita et al., 2018), a key brain region for regulating aspects of cognitive function, particularly memory, and brain inflammation is associated with cognitive impairment (Faraco et al., 2016). Interestingly, evidence is accumulating to suggest that immune cells may directly contribute to cognitive impairment. For example, T cell infiltration in white matter is associated with cognitive decline in aged monkeys (Batterman et al., 2021), and T cell depletion in a mouse model of Alzheimer's disease improves spatial memory (Laurent et al., 2017). Further, in the setting of hypertension, depletion of perivascular macrophages inhibits angiotensin II-induced cognitive impairment (Faraco et al., 2016).

Here, we have infused mice with angiotensin II or aldosterone to test if hypertension can promote brain immune cell infiltration, transcriptomic changes and cognitive impairment. Using hydralazine hydrochloride, we also studied whether development of these effects on the brain following angiotensin II infusion are blood pressure-dependent and reversible.

## 2 Methods

### 2.1 Animals

This study was approved by the La Trobe University Animal Ethics Committee (AEC16-79; AEC16-93). Male C57BL/6J (WT) mice (n = 218; 8–12 weeks old) were obtained from Animal Resource Centre (Canning Vale, Western Australia, Australia). Standard rodent chow and drinking water were provided *ad libitum*. Animals were housed in individually ventilated cages. All animal experiments complied with the ARRIVE guidelines and the National Health and Medical Research Council of Australia code for the care and use of animals for scientific purposes.

### 2.2 Administration of pharmacological agents and measurement of blood pressure

Mice were randomly assigned to treatment groups. Angiotensin II was dissolved in 0.9 % saline and infused S.C. by osmotic minipump (Alzet model 2002 or 2004) at 0.7 mg/kg/d. Control mice for angiotensin II infusion received 0.9 % saline. Aldosterone was dissolved in 87 % propylene glycol, 9 % ethanol and 4 % Milli-Q water and administered S.C. to some mice by osmotic minipump (Alzet model 2002) at 0.72 mg/kg/d. Control mice for aldosterone treatment were infused with 87 % propylene glycol, 9 % ethanol and 4 % Milli-Q water. Mice that were administered vehicle or angiotensin II were maintained on normal drinking water. Mice that were administered aldosterone had their drinking water replaced with 0.9 % saline after minipump implantation.

Mice were anaesthetised with isoflurane (2–4 % inhaled with oxygen, 0.4 L/min) for ∼20 min. Adequacy of anaesthesia was monitored by observing respiration and checking for a reflex response to a toe pinch. An osmotic minipump was implanted S.C. in the mid-scapular region for infusion for 14 or 28 d. At the time of surgery, mice were administered bupivacaine (2.5 mg/kg S.C.) at the surgical site and carprofen (5 mg/kg S.C.). Mice then received carprofen (5 mg/kg S.C.) daily for 2 days after surgery. A subset of angiotensin II-infused mice received hydralazine hydrochloride (50 mg/kg) in their drinking water during the 14 d osmotic minipump infusion, or as an intervention at 2 weeks following the commencement of angiotensin II infusion.

Systolic blood pressure was monitored in conscious mice via tail cuff plethysmography (MC4000 Multichannel system, Hatteras Instruments). Prior to surgery, mice were trained for 1 d (i.e. on day −1) to acclimate to the procedure, and blood pressure was then recorded on days 0 (prior to surgery), 3, 7, 14, 21 and 28 of angiotensin II or aldosterone infusion.

### 2.3 Assessment of neuroinflammation

#### 2.3.1 Flow cytometric analysis of leukocytes in brain

Mice were killed by carbon dioxide inhalation and perfused through the left cardiac ventricle with 0.2 % clexane (400 IU, Sanofi Aventis, Australia) in 0.01 M phosphate-buffered saline (PBS). The left brain hemisphere was harvested for flow cytometry. Brain samples were minced with scissors and digested in PBS (with MgCl_2_ and CaCl_2_) containing a mixture of collagenase type XI (125 U/ml), collagenase type I-S (460 U/ml) and hyaluronidase (60 U/ml) (Sigma-Aldrich, USA) for 30 min at 37 °C. Brain samples were then passed through a 70 μm filter and subjected to a Percoll™ gradient (70 % and 30 % isotonic Percoll) centrifugation from which the layer of mononuclear cells was collected from the interface of the Percoll solutions. Brain cells were stained with an antibody cocktail of anti-CD45 APC-Cy7 (30-F11, Biolegend, USA), anti-CD11b Pacific Blue (M1-70, eBioscience, USA), anti-Ly6G PE-Cy7 (1A8, Biolegend, USA), anti-Ly6C FITC (HK1.4, Biolegend, USA), anti-CD3ε APC (145-2C11, eBioscience, USA), anti-CD4 Alexa Fluor 700 (GK1.5, eBioscience, USA), anti-F4/80 BV711 (BM8, Biolegend, USA) and anti-CD19 PE-Cy5 (6D5, Biolegend, USA), and diluted in PBS containing 0.5 % bovine serum albumin. Samples were analysed via flow cytometry using a CytoFLEX LX flow cytometer (Beckman Coulter, USA) and FlowJo Software (version 10.1, Tree Star Inc, USA, Supplementary Fig. 1). Cell numbers were expressed as total cells per brain hemisphere.

#### 2.3.2 Assessment of brain inflammatory cells

Microglia and astrocytes were localised by staining brain sections for Iba-1 and GFAP, respectively. Whole brains were snap-frozen in liquid nitrogen and stored at −80 °C. Ten μm coronal sections were cut and thaw-mounted onto Superfrost Ultra Plus slides (Thermo Fisher Scientific, USA). Frozen brain sections were air-dried (5 min), fixed with 100 % acetone at 4 °C (10 min) and washed 2 × 3 min with 0.01 M PBS. For Iba-1 staining, sections were incubated with a rabbit anti-Iba-1/AIF-1 primary antibody (1:200; 171985, Cell Signalling Technologies, USA) overnight at 4 °C in antibody diluent. For GFAP staining, sections were incubated with a rabbit anti-GFAP primary antibody (1:1000; ab7260, Abcam, UK) for 1 h at room temperature in antibody diluent. Sections were then washed 2 × 3 min with 0.01 M PBS and then incubated with an Alexa 594 conjugated goat anti-rabbit secondary antibody for 1 h at room temperature (1:400, A11037, ThermoFisher Scientific, MA, USA) and then washed 2 × 3 min with 0.01 M PBS.

Stained sections were mounted with Prolong™ Diamond Antifade mounting medium containing the nuclear stain DAPI (ThermoFisher Scientific, USA) and cover slipped. Sections were stored at 4 °C until imaging. The hippocampus (CA1 and dentate gyrus), cortex and corpus callosum (2 images per animal) were imaged with an Olympus DP73 Camera (Olympus Corporation, Tokyo, Japan) connected to an Olympus BX53 microscope (Olympus Corporation, Tokyo, Japan) at 400× magnification running CellSens Standard Software (version 1.17, Olympus Corporation). Exposure settings, ISO and black balance were kept consistent across all images. Iba-1 positive cells were counted. A custom macro was used to count GFAP positive cells and analyse branches. The macro co-localised fluorescence in green channel with a nucleus and performed a skeleton analysis to assess the branches (Astrocyte Skeleton Analysis v3.1 written by Chad J Johnson, Bioimaging Platform at La Trobe University).

### 2.4 Assessment of blood-brain barrier permeability

Blood-brain barrier permeability was assessed by staining brain sections for endogenous immunoglobulin type G (IgG). Whole brains were snap-frozen in liquid nitrogen and stored at −80 °C. Ten μm coronal sections were cut and thaw-mounted onto Superfrost Ultra Plus slides (Thermo Fisher Scientific, USA). Frozen brain sections were air-dried (5 min), fixed with 100 % acetone at 4 °C (10 min) and washed 3 × 5 min with 0.01 M PBS. For IgG extravasation, sections were incubated with goat anti-mouse IgG (goat anti-mouse IgG (ab150118), Alexa 555, 1:200 dilution) (Abcam, Cambridge, UK) in antibody diluent (3 h), then washed 3 × 5 min with 0.01 M PBS in a dark room. For ZO-1 staining, sections were incubated with a rabbit anti-ZO-1 primary antibody (1:200; 40–2200, ThermoFisher Scientific, MA, USA) for 1 h at room temperature in antibody diluent. Sections were then washed 3 × 5 min with 0.01 M PBS and then incubated with an Alexa 594 conjugated goat anti-rabbit secondary antibody for 1 h at room temperature (1:200, A11037, ThermoFisher Scientific, MA, USA) and then washed 3 × 5 min with 0.01 M PBS.

Stained sections were mounted with VECTASHIELD® mounting medium containing the nuclear stain DAPI (Vector Laboratories, Inc. Burlingame, USA) and cover slipped. Edges were sealed with nail polish and sections were stored at 4 °C until imaging. The hippocampus and cortex (2 images per animal) were imaged with an Olympus DP73 Camera (Olympus Corporation, Tokyo, Japan) connected to an Olympus BX53 microscope (Olympus Corporation, Tokyo, Japan) at 100x and 200× magnification running CellSens Standard Software (version 1.17, Olympus Corporation). Exposure settings, ISO and black balance were kept consistent across all images. Percentage area of staining was analysed using FIJI (National Institute of Health, USA) and the threshold settings were kept consistent across all images.

### 2.5 Gene expression in brain

Messenger RNA (mRNA) expression of inflammatory markers in the brain was determined using TaqMan® real-time PCR. The right brain hemisphere was harvested and snap frozen in liquid nitrogen. Brain hemispheres were sonicated in TRIzol™ (Life Technologies, USA), mixed with chloroform, and centrifuged at 824×*g* for 15 min at 4 °C. The aqueous phase was collected and RNA was extracted using the RNeasy® Mini Kit (Qiagen, USA). RNA was quantified using a NanoDrop One spectrophotometer (Thermo Scientific, USA) and converted to 1st strand cDNA using a High Capacity cDNA RT Kit (Applied Biosystems, USA). Commercially available primers (Applied Biosystems, USA) were used to measure mRNA expression of inflammatory markers, and a house-keeping gene, GAPDH, on a CFX96 Touch Real-Time PCR Detection machine (Bio-Rad, USA). Changes in gene expression were assessed using the delta-delta C_T_ method (Schmittgen and Livak, 2008).

### 2.6 Assessment of working memory

Working memory was assessed by the novel object recognition test (Lueptow, 2017). Mice were acclimated for 2 d by placement in a 40 cm × 40 cm testing box with sawdust on the floor for 10 min. The open field test was performed on day 2 of acclimation. A 30 × 30 cm zone was set up in the middle of the box (defined as the inner zone) and a 5 cm wide zone around the edges of the box (outer zone). Time in the inner zone was tracked using automated software (Ethovision XT, Noldus Information Technology BV, Wageningen, The Netherlands). On the day of testing, the mouse was placed in the same box with two identical objects for 10 min. One h later, the mouse was placed back in the same box for 5 min with one of the familiar objects replaced by a novel object. The cumulative minimum interaction time during the retention phase was 5 s (Faraco et al., 2016; Diaz-Otero et al., 2018). The mouse's interactions with the objects (nose point entering a zone 2 cm wide around the object) were tracked using Ethovision XT.

### 2.7 RNA sequencing

RNA sequencing was performed as previously described (Baik et al., 2021). Brain hemispheres were snap frozen in liquid nitrogen. In a separate cohort of mice, the hippocampus was dissected from the brain and snap frozen in liquid nitrogen. Brain hemispheres and hippocampi were sonicated in TRIzol™ (Life Technologies, USA), mixed with chloroform, and centrifuged at 824×*g* for 15 min at 4 °C. The aqueous phase was collected and RNA was extracted using the RNeasy® Micro Kit (Qiagen, USA). RNA was quantified using a NanoDrop One spectrophotometer (Thermo Scientific, USA) and then stored at −80 °C. The RNA samples were shipped to NovogeneAIT Genomics (Singapore) for cDNA library preparation and RNA sequencing. mRNA was purified from total RNA using poly-T oligo-attached magnetics. mRNA was converted to cDNA and purified using AMPure XP Beads (Beckman Coulter Life Sciences, USA). cDNA libraries were acquired by PCR amplification. High-throughput sequencing was conducted using the HiSeqTM 2500 platform (Illumina, USA). The results were mapped to the Ensembl-released mouse genome sequence and annotation. Differential expression analysis was conducted using the DESeq R Package V.1.10.1 and P-values were adjusted using the Banjamini and Hochberg's approach for controlling the false discovery rate. Genes were considered differentially expressed if the adjusted P-value was less than 0.05. R package heatmap 3 and log2Fold-Change output from EdgeR V.3.2.4 were used to create heatmaps for differentially expressed genes.

A gene set enrichment analysis (GSEA), which takes a pre-ranked list of all expressed genes, was used to identify gene ontologies with concordant differences in gene expression. Log_2_-fold changes for all expressed genes were obtained using the DESeq2 package (v1.40.2) (Love et al., 2014). The clusterProfiler (v4.4.4) (Wu et al., 2021; Yu et al., 2012) package was used to obtain gene set terms from the Molecular Signature Database from the msigdbr package (v7.5.1) (Liberzon et al., 2015; Subramanian et al., 2005) with a minimum gene set size and maximum gene set size of 25 and 500 genes, respectively. Gene ontology (GO) results were then visualised using the enrichplot package (v1.18.3).

### 2.8 Statistical analysis

Results are expressed as mean ± S.E.M. Power calculations were performed using G∗Power. Statistical analyses between groups were performed using Student's unpaired *t*-test, one-sample *t*-test, or a one- or two-way ANOVA followed by a Tukey's or Sidak's post-hoc test, as appropriate. P < 0.05 was considered to be significant. RNA sequencing data were analysed using R Studio. GraphPad Prism software (version 8.0, GraphPad Software Inc., USA) was used to perform all other statistical analyses.

## 3 Results

### 3.1 Infusion of angiotensin II or aldosterone promotes immune cell infiltration in the brain

**Fig. 1:**
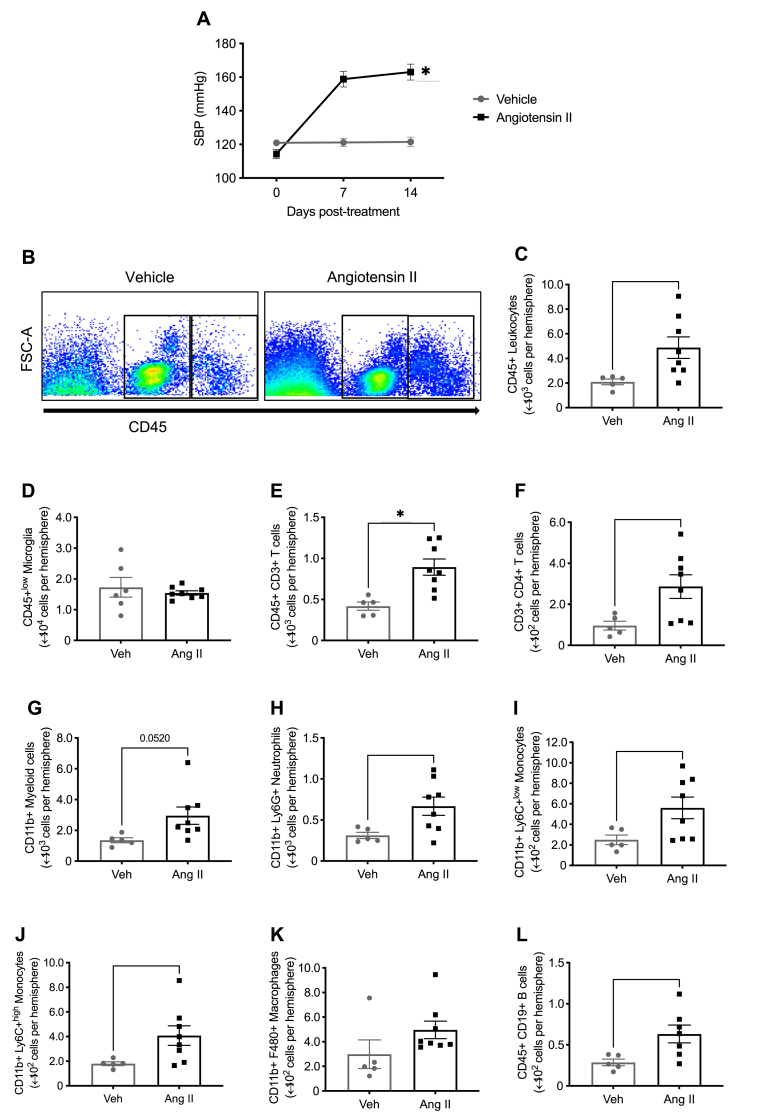
**Angiotensin II infusion promotes immune cell infiltration in the brain. A:** Angiotensin II-induced hypertension (n = 6–8). All data are mean ± S.E.M. ∗P < 0.05. Two-way ANOVA with Tukey's test. **B:** Representative flow cytometry dot plots showing gating strategy for microglia (CD45^+^ low) and total leukocytes (CD45^+^ high) from the brains of mice infused with vehicle or angiotensin II. The effect of angiotensin II infusion on **C:** CD45^+^ leukocytes, **D:** CD45+^low^ microglia, **E:** CD3^+^ T cells, **F:** CD4^+^ T cells, **G:** CD11b + myeloid cells, **H:** Ly6G + neutrophils, **I:** Ly6C + ^low^ monocytes, **J:** Ly6C + ^high^ monocytes, **K:** F4/80+ macrophages and **L:** CD19^+^ B cells in the brain (n = 6–8). All data are mean ± S.E.M. ∗P < 0.05. Student's unpaired *t*-test.

**Fig. 2:**
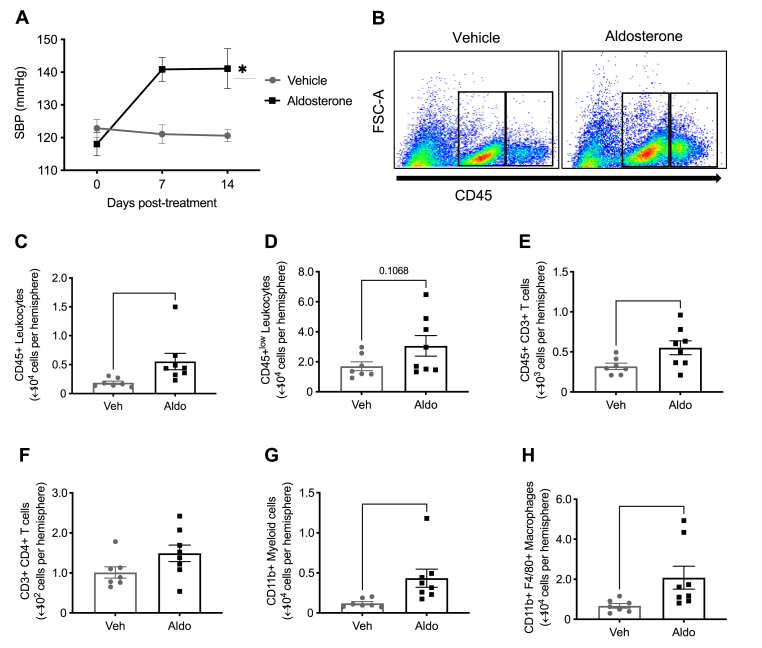
**Aldosterone infusion promotes immune cell infiltration in the brain. A:** Aldosterone-induced hypertension (n = 8). All data are mean ± S.E.M. ∗P < 0.05. Two-way ANOVA with Tukey's test. **B:** Representative flow cytometry dot plots showing gating strategy for microglia (CD45^+^ low) and total leukocytes (CD45^+^ high) from the brains of mice infused with vehicle or aldosterone. The effect of aldosterone infusion on **C:** CD45^+^ leukocytes, **D:** CD45+^low^ microglia, **E:** CD3^+^ T cells, **F:** CD4^+^ T cells, **G:** CD11b + myeloid cells and **H:** F4/80+ macrophages in the brain (n = 8). All data are mean ± S.E.M. ∗P < 0.05. Student's unpaired *t*-test.

Angiotensin II infusion caused a marked increase in systolic blood pressure (by ∼40 mmHg, Fig. 1A). Elevation of blood pressure increased the accumulation of circulating CD45^+^ leukocytes (Fig. 1B and C; gating strategy for other leukocytes are shown in the Supplementary Fig. 1) in the brain. However, there was no effect on the number of microglia (CD45^low^; Fig. 1D). Angiotensin II infusion increased the accumulation of T cells (Fig. 1E and F), myeloid cells (Fig. 1G), neutrophils (Fig. 1H), monocytes (Fig. 1I and J) and B cells (Fig. 1L) in the brain. There was also a tendency for more macrophages (Fig. 1K; *P* = 0.15). Similarly, aldosterone infusion caused elevation of systolic blood pressure (by ∼20 mmHg, Fig. 2A) and accumulation of leukocytes (Fig. 2B and C) in the brain. However, the number of microglia (CD45^low^; Fig. 2D) was not altered by aldosterone. Furthermore, aldosterone promoted the accumulation of T cells (Fig. 2E), myeloid cells (Fig. 2G) and macrophages (Fig. 2H) in the brain. Representative flow cytometry plots are shown in Supplementary Figs. 2–4.

### 3.2 Hydralazine prevents angiotensin II-induced hypertension, cognitive dysfunction and blood brain barrier breakdown

**Fig. 3:**
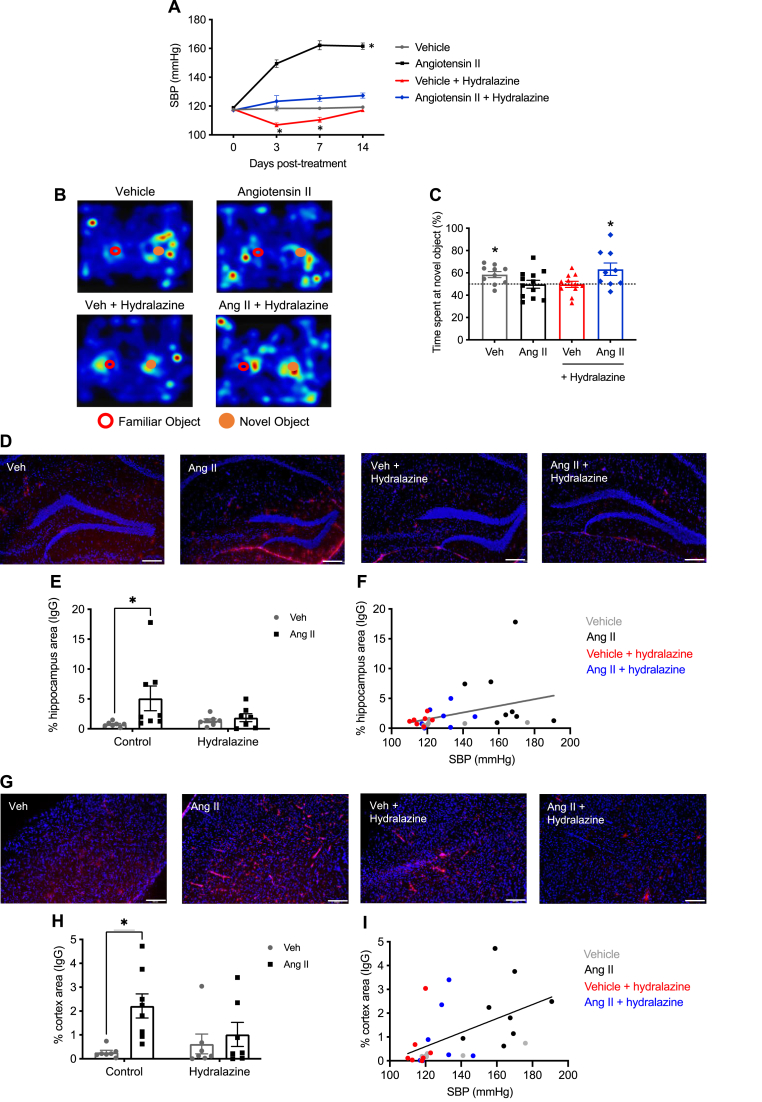
**Angiotensin II infusion promotes cognitive impairment and blood-brain barrier breakdown which is prevented by co-treatment with hydralazine. A:** The effect of hydralazine on blood pressure in mice infused with vehicle or angiotensin II (n = 7–8). All data are mean ± S.E.M. ∗P < 0.05 vs vehicle. Two-way ANOVA with Tukey's test. **B:** Representative heatmap plots showing interaction between familiar and novel objects in mice infused with vehicle, angiotensin II, vehicle + hydralazine and angiotensin II + hydralazine. **C:** Effect of angiotensin II infusion and co-treatment of hydralazine on recognition memory (n = 9–12). All data are mean ± S.E.M. ∗P < 0.05. One-sample *t*-test vs. 50 %. **D:** Representative images showing IgG deposition (Alexa Flour 555, red) in hippocampus of mice infused with vehicle, angiotensin II, vehicle + hydralazine and angiotensin II + hydralazine. Nuclei are identified by DAPI counter-stain (blue). Scale bar = 200 μm. **E:** Effect of angiotensin II infusion and co-treatment of hydralazine on IgG deposition in the hippocampus (n = 7–8). All data are mean ± S.E.M. ∗P < 0.05. Two-way ANOVA with Sidak's test. **F:** Correlation between IgG deposition in hippocampus and systolic blood pressure (R^2^ = 0.13, P = 0.05). **G:** Representative images showing IgG deposition (Alexa Flour 555, red) in cortex of mice infused with vehicle, angiotensin II, vehicle + hydralazine and angiotensin II + hydralazine. Nuclei are identified by DAPI counter-stain (blue). Scale bar = 200 μm. **H:** Effect of angiotensin II infusion and co-treatment of hydralazine on IgG deposition in the cortex (n = 7–8). All data are mean ± S.E.M. ∗P < 0.05. Two-way ANOVA with Sidak's test. **I:** Correlation between IgG deposition in cortex and systolic blood pressure (R^2^ = 0.26, P < 0.05). (For interpretation of the references to colour in this figure legend, the reader is referred to the Web version of this article.)

Co-administration of hydralazine blunted the pressor response to angiotensin II and reduced systolic blood pressure in vehicle-infused mice at days 3 and 7 (Fig. 3A). In the open field test, vehicle-infused mice treated with hydralazine spent a significantly lower percentage of time in the inner zone compared with angiotensin II + hydralazine treated mice (Supplementary Fig. 5A). The percentage of time in the inner zone was comparable between all other groups (Supplementary Fig. 5B). In the novel object recognition test to assess working memory, there were no differences between groups in the cumulative amount of time spent interacting with the objects during the retention phase (Supplementary Fig. 5B). Vehicle-infused mice spent a majority of time interacting with the novel object (Fig. 3B and C) whereas angiotensin II-infused mice did not discriminate between novel and familiar objects (Fig. 3B and C). Importantly, no such cognitive dysfunction was evident in angiotensin II-infused mice co-treated with hydralazine (Fig. 3B and C). There was also evidence of memory impairment in a minority of the vehicle-infused mice treated with hydralazine (Fig. 3C).

Angiotensin II increased IgG deposition in the hippocampus (Fig. 3D–F) and cortex (Fig. 3G–I), and this was prevented by co-treatment with hydralazine. Across all mice, IgG deposition was positively correlated in hippocampus (R^2^ = 0.13, P < 0.05, Fig. 3F) and cortex (R^2^ = 0.26, P < 0.05, Fig. 3I) with systolic blood pressure. We did not observe any change in the gene expression of claudin-5 (*Cldn5*) or zonula occludens 1 (*Tjp1;* ZO-1) between groups based on our RNA sequencing data (Supplementary Fig. 6A–B). Furthermore, we did not observe any change in ZO-1 immunofluorescence staining in the hippocampus or cortex between groups (Supplementary Fig. 7A and B).

### 3.3 Hydralazine prevents angiotensin II-induced immune cell infiltration and neuroinflammation

**Fig. 4:**
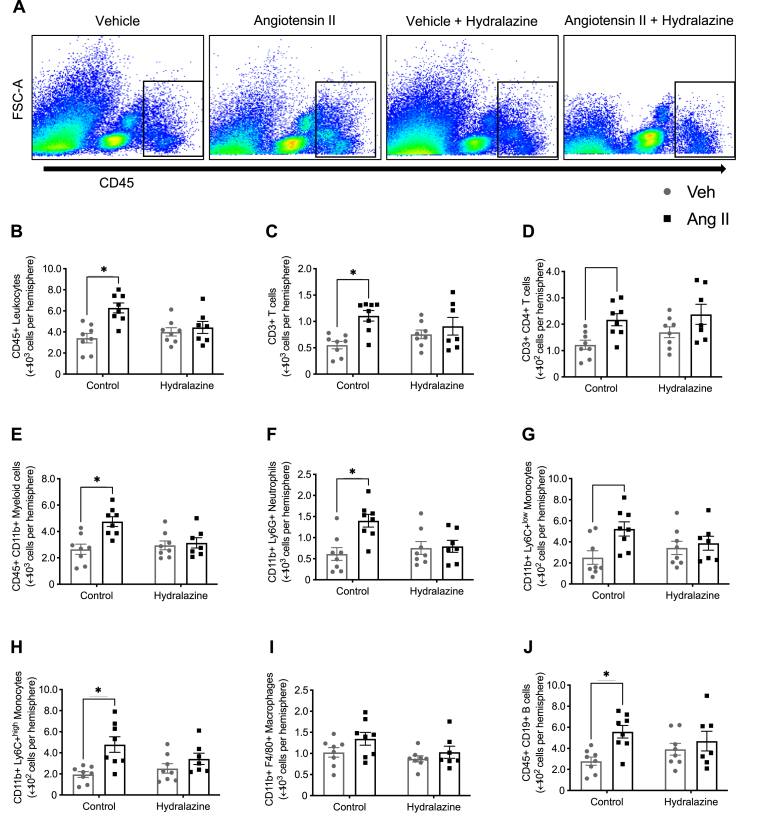
**Angiotensin II-induced increase in brain immune cell infiltration is blood pressure-dependent. A:** Representative flow cytometry dot plots showing gating strategy for total leukocytes (CD45^+^ high) from the brains of mice infused with vehicle, angiotensin II, vehicle + hydralazine and angiotensin II + hydralazine. The effect of angiotensin infusion and co-treatment with hydralazine on **B:** CD45^+^ leukocytes, **C:** CD3^+^ T cells, **D:** CD4^+^ T cells, **E:** CD11b + myeloid cells, **F:** Ly6G + neutrophils, **G:** Ly6C + ^low^ monocytes, **H:** Ly6C + ^high^ monocytes, **I:** F4/80+ macrophages and **J:** CD19^+^ B cells in the brain (n = 7–8). All data are mean ± S.E.M. ∗P < 0.05. Two-way ANOVA with Tukey's test.

Co-treatment with hydralazine prevented angiotensin II-induced increases in CD45^+^ leukocytes in the brain (Fig. 4A and B), including T cells (Fig. 4C and D), myeloid cells (Fig. 4E), neutrophils (Fig. 4F), monocytes (Fig. 4G and H) and B cells (Fig. 4J). Numbers of macrophages were not different among all groups (Fig. 4I).

**Fig. 5:**
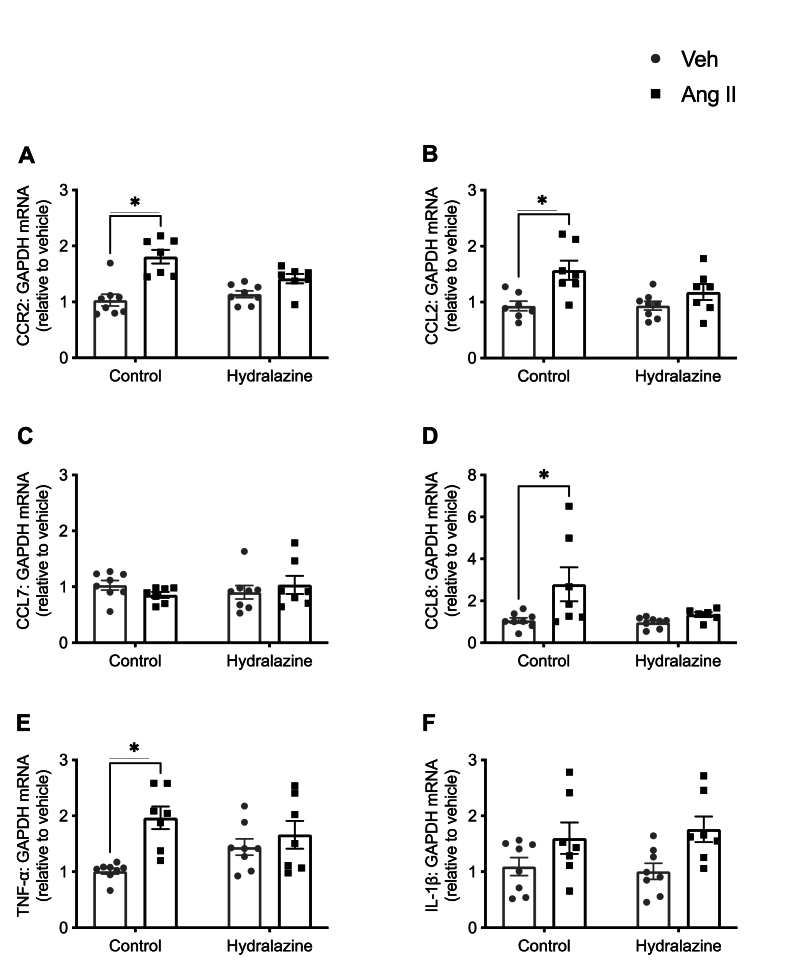
**Angiotensin II-induced increase in neuroinflammation is blood pressure-dependent.** The effect of angiotensin infusion and co-treatment with hydralazine on mRNA expression of **A:** chemokine (C-C motif) receptor 2 (CCR2), **B:** chemokine (C-C motif) ligand (CCL) 2, **C:** CCL7, **D:** CCL8, **E:** tumour necrosis factor-α, and **F:** interleukin-1β in the brain (n = 7–8). All data are mean ± S.E.M. ∗P < 0.05. Two-way ANOVA with Tukey's test.

Angiotensin II promoted gene expression of chemokine (C-C motif) receptor 2 (CCR2) (Fig. 5A), chemokine (C-C motif) ligand 2 (CCL2) (Fig. 5B), CCL8 (Fig. 5D) and TNF-α (Fig. 5E) but had no effect on CCL7 (Fig. 5C) and interleukin-1β (Fig. 5F). Co-treatment with hydralazine prevented the angiotensin II-induced increases in gene expression of CCR2 (Fig. 5A), CCL2 (Fig. 5B), CCL8 (Fig. 5D) and TNF- α (Fig. 5E).

Angiotensin II did not affect the number of Iba-1 positive cells in the CA1 (Supplementary Fig. 8), dentate gyrus (Supplementary Fig. 9), cortex (Supplementary Fig. 10) and corpus callosum (Supplementary Fig. 11) compared to vehicle. Angiotensin II also did not affect the number of GFAP positive cells and branches per cell in the CA1 (Supplementary Fig. 12), dentate gyrus (Supplementary Fig. 13) and corpus callosum (Supplementary Fig. 14) compared to vehicle. GFAP positive cells were not observed in the cortex (data not shown).

### 3.4 Hydralazine modulates expression of genes in the brain

**Fig. 6:**
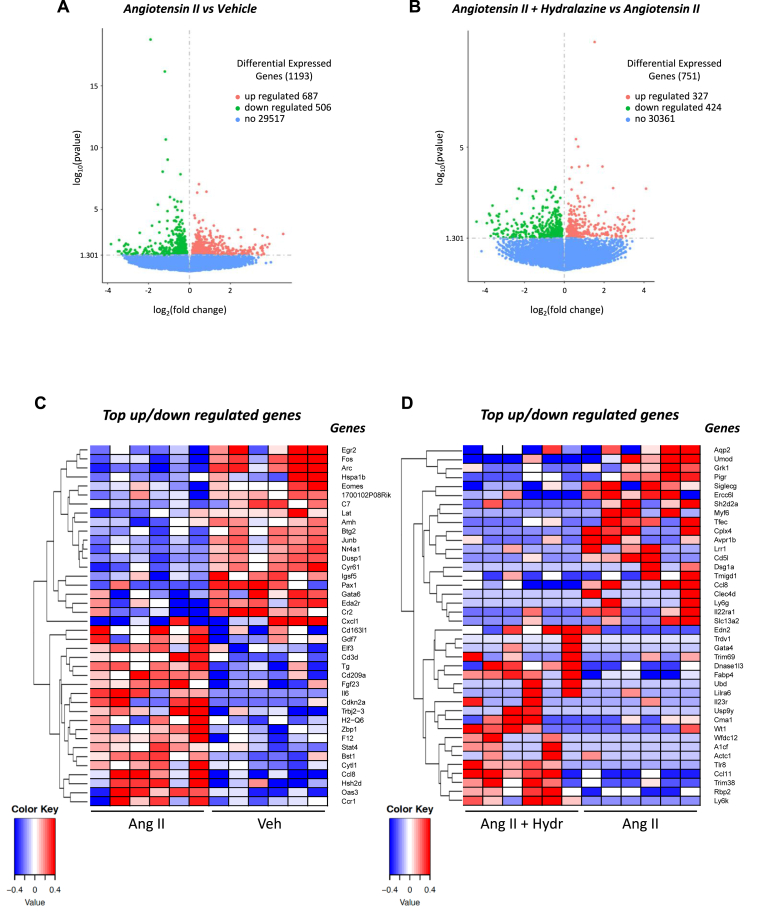
**Angiotensin II infusion promotes transcriptomic changes in the brain which is modulated by treatment with hydralazine.** Volcano plot of differentially expressed genes in the brains of mice infused with **A:** vehicle vs angiotensin II or **B:** angiotensin II vs angiotensin II + hydralazine. The threshold of differential expression is p-value <0.05. The horizontal axis is the log2 fold change of genes. The vertical axis is statistical significance scaled as -log 10 p-value. Each dot represents an individual gene (blue: no significant difference; red: upregulated expression; green: downregulated expression). The top upregulated and downregulated genes in brains of mice infused with **C:** vehicle vs angiotensin II or **D:** angiotensin II vs angiotensin II + hydralazine. Upregulated genes in red and downregulated genes in blue. The colour scale represents the log10 (average FPKM + 1) value. (For interpretation of the references to colour in this figure legend, the reader is referred to the Web version of this article.)

Bulk RNA sequencing was performed on the brains of mice infused with vehicle, angiotensin II or angiotensin II + hydralazine. When comparing mice infused with angiotensin II alone versus vehicle alone, there were 1193 differentially expressed genes in the whole brain (687 upregulated and 506 downregulated; Fig. 6A). There were 751 differentially expressed genes (327 upregulated and 424 downregulated) when comparing mice infused with angiotensin II alone versus angiotensin II + hydralazine in the whole brain (Fig. 6B).

We next performed further analysis focussing on genes related to inflammation. In this analysis, the genes most upregulated by angiotensin II included cd163 molecule like 1 (*Cd163l1*), growth differentiation factor 7 (*Gdf7*), e74 like ETS transcription factor 3 (*Elf3*), cd3d delta subunit of t cell receptor complex (*Cd3d*), cd209 molecule (*Cd209*), fibroblast factor growth factor 23 (*Fgf23*), interleukin 6 (*Il6*), t cell receptor beta joining 2–3 (*Trbjj2-3)*, histocompatibility 2, Q region locus 6 (*H2-q6*), z-dna binding protein 1 (*Zbp1*), coagulation factor XII (*F12*), CC motif chemokine ligand 8 (*Ccl8*), hematopoietic SH2 domain containing (*Hsh2d*) and CC motif chemokine receptor 1 (*Ccr1*) (Fig. 6C). The top downregulated genes following infusion with angiotensin II compared to vehicle included early growth response 2 (*Egr2*), fos proto-oncogene, AP-1 transcription factor subunit (*Fos*) activity regulated cytoskeleton associated protein (*Arc*), complement c7 (*C7*), BTG anti-proliferation factor 2 (*Btg2*)*,* jun B proto-oncogene (*Junb*), nuclear receptor subfamily 4 group A member (*Nr4a1*), dual specificity phosphatase 1 (*Dusp1*) and complement receptor 2 (*Cr2*) (Fig. 6C).

The genes most upregulated by angiotensin + hydralazine compared to angiotensin II in the brain included endothelin 2 (*Edn2),* T cell receptor delta variable 1 (*Trdv1*), ubiquitin D *(Ubd),* interleukin 23 receptor *(Il23r),* toll like receptor 8 *(Tlr8),* tripartite motif containing 38 *(Trim 38),* C-C motif chemokine ligand 11 (*Ccl11*) and lymphocyte antigen 6 family member K (*Ly6k*) (Fig. 6D). The top downregulated genes by angiotensin II + hydralazine compared to angiotensin II in the brain included aquaporin-2 (*Aqp2*), uromodulin (*Umod*), polymeric immunoglobulin receptor (*Pigr*), sialic acid binding Ig like lectin 10 (*Siglecg*), Sh2 domain containing 2a (*Sh2d2a*), C-C motif chemokine ligand 8 (*Ccl8*), lymphocyte antigen 6 family member G (*Ly6g)*, interleukin 22 receptor subunit alpha 1 (*Il22ra*) and C-type lectin domain family 4 member D (*Clec4d*) (Fig. 6D).

**Fig. 7:**
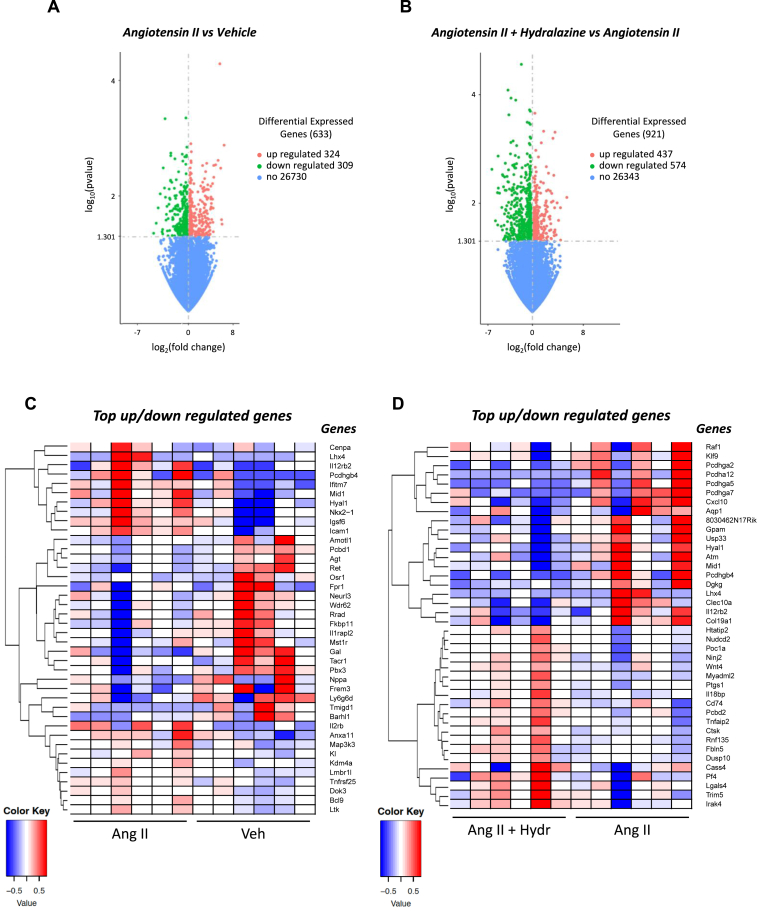
**Angiotensin II infusion promotes transcriptomic changes in the hippocampus which is modulated by treatment with hydralazine.** Volcano plot of differentially expressed genes in the hippocampus of mice infused with **A:** vehicle vs angiotensin II or **B:** angiotensin II vs angiotensin II + hydralazine. The threshold of differential expression is p-value <0.05. The horizontal axis is the log2 fold change of genes. The vertical axis is statistical significance scaled as -log 10 p-value. Each dot represents an individual gene (blue: no significant difference; red: upregulated expression; green: downregulated expression). The top upregulated and downregulated genes in hippocampus of mice infused with **C:** vehicle vs angiotensin II or **D:** angiotensin II vs angiotensin II + hydralazine. Upregulated genes in red and downregulated genes in blue. The colour scale represents the log10 (average FPKM + 1) value. (For interpretation of the references to colour in this figure legend, the reader is referred to the Web version of this article.)

When comparing mice infused with angiotensin II alone versus vehicle alone, there were 633 differentially expressed genes (324 upregulated and 309 downregulated) specifically in the hippocampus (Fig. 7A). There were 921 differentially expressed genes (347 upregulated and 574 downregulated) in the hippocampus when comparing mice infused with angiotensin II alone versus angiotensin II + hydralazine (Fig. 7B).

Further analysis revealed that the genes most upregulated by angiotensin II compared to vehicle in the hippocampus included interleukin 12 receptor subunit beta 2 (*Il12rb2*), protocadherin gamma subfamily b4 (*Pcdhgb4*), interferon induced transmembrane protein 7 (*Ifitm7*), hyaluronidase 1 (*Hyal1*), immunoglobulin superfamily member 6 (*Igsf6*), intercellular adhesion molecule 1 (*Icam1*), interleukin 2 receptor subunit beta (*Il2rb*), annexin A11 (*Anxa11*), lysine demethylase 4a (*Kdm4a*), TNF receptor superfamily member 25 (*Tnfrsf25*), docking protein 3 (*Dok3*) and B cell CLL/lymphoma 9 protein (*Bcl9*) (Fig. 7C). The top downregulated genes following infusion with angiotensin II compared to vehicle included pterin-4 alpha-carbinolamine dehydratase (*Pcbd1*), angiotensinogen (*Agt*), odd-skipped related transcription factor 1 (*Osr1*), formyl peptide receptor 1 (*Fpr1*), WD repeat domain 62 (*Wdr62*), FKBP prolyl isomerase 11 (*Fkbp11*), interleukin 1 receptor accessory protein 2 (*Il1rapl2*), macrophage stimulating 1 receptor (*Mst1r*), tachykinin receptor 1 (*Tacr1*), natriuretic peptide A (*Nppa*) and BarH like homeobox 1 (*Barhl1*).

The genes most upregulated by angiotensin II + hydralazine compared to angiotensin II in the hippocampus included ninjurin 2 (*Ninj2*), wnt family member 4 (*Wnt 4*), prostaglandin-endoperoxide synthase 1 (*Ptgs1*), interleukin-18 binding protein (*Il18bp*), cd74 molecule (*Cd74*), tumour necrosis factor alpha-induced protein 2 (*Tnfaip2*), cathepsin k (*Ctsk*), dual specificity phosphatase 10 (*Dusp10*), platelet factor 4 (*Pf4*), tripartite motif containing 4 (*Trim4i*) and interleukin 1 receptor associated kinase 4 (*Irak4*) (Fig. 7D). The top downregulated genes by angiotensin II + hydralazine compared to angiotensin II in the hippocampus included raf-1 proto-oncogene, serine/threonine kinase (*Raf 1*), KLF transcription factor 9 (*Klf9*), protocadherin gamma subfamily A, 2 (*Pcdhga2*), C-X-C motif chemokine ligand 10 (*Cxcl10*), aquaporin 1 (*Aqp1*), ubiquitin specific peptidase 33 (*Usp 33*), hyaluronidase 1 (*Hyal1*), midline 1 (*Mid 1*), protocadherin gamma subfamily b4 (*Pcdhgb4*), diacylglycerol kinase gamma (*Dgkg*), C-type lectin domain containing 10a (*Clec10a*), interleukin 12 receptor subunit beta 2 (*Il12rb2*) and collagen type XIX alpha 1 chain (*Col19a1*).

We next performed GSEA to identify up or down regulated GO terms. In the whole brain, GO terms relating to the adaptive immune response, antigen binding and formation of immunoglobulin complexes were upregulated in angiotensin II compared to vehicle treated mice (Supplementary Fig. 15A).

In the hippocampus of angiotensin II compared to vehicle treated mice, GO terms including intermediate filament process and organisation, cytoskeleton structure and epidermis development were downregulated (Supplementary Fig. 15B). In the hippocampus of angiotensin II + hydralazine compared to angiotensin II treated mice, GO terms related to homophilic cell adhesion molecules were downregulated while GO terms related to ribosomal structure were upregulated (Supplementary Fig. 15C).

### 3.5 Intervention with hydralazine during established hypertension reduces blood pressure but not brain inflammation or cognitive impairment

**Fig. 8:**
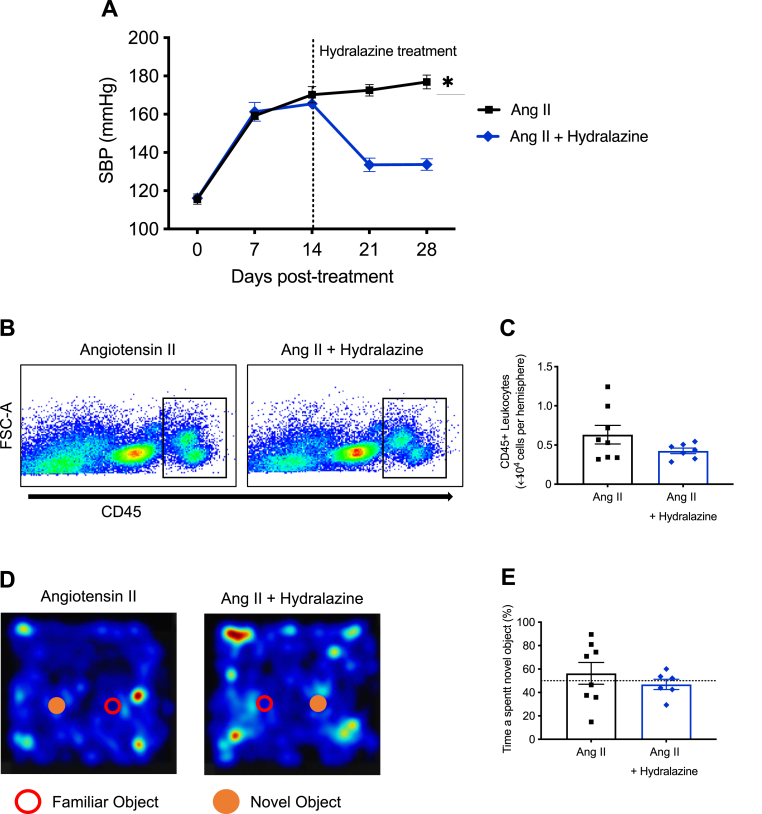
**Intervention with hydralazine does not reverse angiotensin II-induced immune cell infiltration in the brain and cognitive impairment. A:** Angiotensin II-induced hypertension and effect of intervention with hydralazine (n = 7–8). All data are mean ± S.E.M. ∗P < 0.05. Two-way ANOVA with Tukey's test. **B:** Representative flow cytometry dot plots showing gating strategy for total leukocytes (CD45^+^ high) from the brains of mice infused with angiotensin II and angiotensin II + hydralazine. **C:** The effect of intervention with hydralazine on angiotensin II-induced leukocyte infiltration in the brain (n = 7–8). All data are mean ± S.E.M. **D:** Representative heatmaps showing interaction between familiar and novel objects in mice infused with angiotensin II and angiotensin II + hydralazine. **E:** The effect of intervention with hydralazine on angiotensin II-induced cognitive impairment (n = 7–8). All data are mean ± S.E.M.

Angiotensin II infusion increased systolic blood pressure which was sustained for 28 days (Fig. 8A). In some mice, intervention with hydralazine from day 14–28 of angiotensin II infusion reduced blood pressure to almost baseline (Fig. 8A). However, at day 28 there was no significant difference between groups in either immune cell numbers in the brain (Fig. 8B–C) or cognitive function (Fig. 8D–E). We did not observe any difference in the percentage of time spent in the inner zone (open field test), or cumulative interaction time with the novel and familiar objects between groups (Supplementary Fig. 16A–B).

## 4 Discussion

There are several novel findings of this study. First, neuroinflammation, IgG deposition, transcriptomic changes and cognitive impairment during hypertension are dependent on the degree of elevated blood pressure. Second, treatment of hypertensive mice with hydralazine for two weeks does not reverse brain inflammation or cognitive impairment. Our findings are consistent with the concept that chronic hypertension promotes brain inflammation and memory impairment, but these effects may not be readily reversible by lowering blood pressure.

Chronic hypertension is known to cause brain injury, and indeed infusion with angiotensin II or aldosterone can promote cerebrovascular dysfunction (Chrissobolis et al., 2014) and oxidative stress (Dinh et al., 2016). Hypertension may also promote leukocyte infiltration in blood pressure-regulating organs including the kidneys (Dinh et al., 2021; Krishnan et al., 2019) and blood vessels (Chan et al., 2015; Wei et al., 2014; Dinh et al., 2024). We and others have reported that angiotensin II infusion promotes T cell infiltration in the brain (Wei et al., 2014; Don-Doncow et al., 2019), and here we show that there are increases in several immune cell subsets in addition to T cells in the brain during two models of experimental hypertension. While the specific immune cell subtype(s) responsible to the deleterious effects of hypertension on the brain remain to be elucidated, it is likely that the several immune cell subtypes act in a coordinated manner to promote brain injury (Guzik et al., 2024).

Under normotensive conditions, circulating cells and numerous molecules are prevented from entering the brain by the blood-brain barrier, but chronic hypertension can cause blood-brain barrier breakdown, allowing entry to the brain of a variety of factors such as immune cells. IgG is one such class of circulating proteins that do not normally penetrate the blood-brain barrier, but our finding of significant IgG deposition in the hippocampus and cortex during hypertension is indicative of hypertension-induced blood-brain barrier dysfunction. These findings are analogous to reports that peripheral immune infiltrate the brain stroke (Qian et al., 2018) and in Alzheimer's disease (Zenaro et al., 2015). The increased BBB permeability occurred despite no changes in expression of select genes and proteins that regulate BBB integrity. Taken together, our finding of increased IgG deposition despite no changes in some BBB related genes/proteins may suggest that the function of these key molecules is disrupted, leading to increased BBB permeability. In our study, oral administration of the anti-hypertensive agent hydralazine prevented angiotensin II-induced hypertension as well as IgG deposition and immune cell infiltration, suggesting that these latter changes were also blood pressure-dependent. Hydralazine also prevented angiotensin II-induced expression of *Ccr2*, and its ligands *Ccl2* and *Ccl8*, consistent with *Ccr2*-mediated immune cell infiltration as a key aspect of brain inflammation during hypertension. We note that hydralazine has been reported to have anti-oxidant, anti-inflammatory, anti-apoptotic effects in addition to its vasodilatory actions (Chang and Chen, 2022). These effects of hydralazine would protect against many of the mechanisms by which a hypertensive stimulus (such as angiotensin II) would increase blood pressure and end-organ damage. Further studies are required to determine whether the protective effects of hydralazine are solely dependent on its blood pressure lowering effects or whether the anti-oxidant, anti-inflammatory and anti-apoptotic effects of hydralazine are also key mechanisms.

Hypertension is a major risk factor for cognitive impairment in humans, and our data are consistent with such a relationship in mouse models (Csiszar et al., 2013; Foulquier et al., 2018). Importantly, our study provides direct evidence that angiotensin II-induced cognitive impairment is indeed blood pressure-dependent. There are a number of ways in which hypertension could cause brain injury and cognitive impairment, such as through disruption of cerebral artery structure and function which could ultimately result in cerebral small-vessel disease, ischemic white matter lesions and vascular dementia (Iadecola et al., 2016; Verhaaren et al., 2013). Indeed, higher systolic blood pressure is associated with faster decline in cognition (Levine et al., 2019), and poorer cognition is associated with more severe hypertension (Muela et al., 2017). While results from the Systolic Blood Pressure Intervention Trial (SPRINT) substudy Memory and Cognition in Decreased Hypertension (SPRINT MIND) suggest that intensive reduction in systolic blood pressure (<120 mmHg) decreases the risk of developing mild cognitive impairment but not dementia in patients compared to standard reduction in systolic blood pressure (<140 mmHg) The SPRINT MIND Investigators, (Williamson et al., 2019), there were no apparent effects on brain inflammation and cognitive deficit after just two weeks of hypertension reversal in our study in mice.

Accumulating evidence has suggested inflammation to be a mediator of cognitive impairment (Skelly et al., 2018). Vascular inflammation is one mechanism by which hypertension may alter the structure and function of cerebral blood vessels through processes such as vascular remodelling (Shenoy et al., 2018) and fibrosis (Aldrich and Kielian, 2011). Furthermore, inflammation and immunity are features of cerebral small vessel disease (Fu and Yan, 2018). Since anti-hypertensive therapy with hydralazine prevented both angiotensin II-induced brain inflammation and cognitive impairment, our data are consistent with the concept that hypertension-induced brain inflammation leads to cognitive impairment. Indeed, targeting inflammation is reported to reduce cognitive impairment during hypertension (Faraco et al., 2016; Jalal et al., 2015) and other cognitive disorders (Ni et al., 2018; Fu et al., 2019). While it has been suggested that microglia and astrocytes may contribute to brain inflammation, we did not observe any changes to these cell types at the timepoint investigated.

Bulk RNA sequencing showed that angiotensin II infusion upregulated genes in the brain involved in biological processes such as inflammation (*Gdf7, Elf3*), immune cell migration (*Ccl8, Ccr1*), macrophage activity (*Cd163l1, Cd209*), T cell signalling and proliferation (*Cd3d, Trbjj2-3, H2-q6, Hsh2d, Il6*), PANoptosis (*Zbp1*), fibrosis (*Fgf23*) and thrombosis (*F12*). This supports our flow cytometry data that angiotensin II activated pathways that promote immune cell migration and neuroinflammation. Consistent with the differential gene analysis, we identified GO terms relating to adaptive immune response, antigen binding and immunoglobulin complexes were upregulated. Together, these data suggest that angiotensin II upregulates key inflammatory processes in the brain. Angiotensin II downregulated genes involved in biological processes such as memory consolidation (*Arc*), anti-inflammatory (*Dusp1*), T regulatory cell development (*Junb*), macrophage differentiation (*Egr2*), negative regulation of microglia proliferation (*Btg2*), inhibition of apoptosis (*Nr4a1*) and the complement pathway (*C7, Cr2*). *Arc* is a master regulator of long-term memory formation (Plath et al., 2006), hence, downregulation of *Arc* may have contributed to the cognitive decline observed in angiotensin II-infused mice.

In the hippocampus, a key brain region for learning and memory, angiotensin II promoted upregulation of genes involved in inflammation (*Igsf6, Hyal1*), T cell differentiation and activity (*Il12rb2, Ifitm7, Il2rb, Tnfrsf25*), microglia regulation (*Kdm4a, Dok3*), cell adhesion (*Pcdhgb4, Icam1*) and apoptosis (*Anxa11, Bcl9*). Immune cells specifically in the hippocampus were not analysed in our study but the data are suggestive of increased immune cell infiltration as there was upregulation of the cell adhesion molecule, *Icam1,* and evidence of BBB injury*. Il12rb2, Ifitm7, Il2rb* and *Tnfrsf25* are involved in T cell differentiation and proliferation, consistent with a T cell response in the hippocampus. We showed an increased brain infiltration of T cells, which have been reported to promote cognitive impairment in a mouse model of Alzheimer's disease (Machhi et al., 2021). Hence, the presence of T cells in the hippocampus may have contributed to the development of cognitive impairment.

Angiotensin II downregulated genes involved in anti-inflammatory pathways such as *Dusp1* and *Btg2*. Overexpression of *Dusp1* attenuates inflammation in a mouse model of cardiomyopathy (Tan et al., 2022) while deficiency of *Dusp1* has been shown to exacerbate inflammation in a mouse model of septic peritonitis (Hammer et al., 2010). Deficiency of *Btg2* has been found to increase activation of microglia and impair spatial learning ability in a mouse model of chronic cerebral hypoperfusion (Suzuki et al., 2021). Downregulation of these genes thus likely contributed to a pro-inflammatory response to angiotensin II. Co-treatment with angiotensin II + hydralazine caused upregulation of *Dusp1* and *Btg2* suggesting that hydralazine reduced neuroinflammation partly through these pathways. Co-administration of angiotensin II and hydralazine also promoted downregulation of markers of immune cells and their activity (*Sh2d2a, Ly6g*), immune cell migration (*Ccl8*) and immunoglobulin binding (*Umod, Pigr)* consistent with protective effects of hydralazine on neuroinflammation. Interestingly, administration of hydralazine with angiotensin II reversed the upregulation of *Il12rb2* in the hippocampus caused by angiotensin II alone. As mentioned, *Il12rb2* is involved in T cell differentiation and proliferation and so the resulting reduction in hippocampal neuroinflammation by hydralazine may have contributed to the preservation of cognitive function during angiotensin II infusion, potentially as an indirect effect of blood pressure reduction.

It is noteworthy that, while we found that development of brain inflammation and cognitive impairment during hypertension were blood pressure-dependent, intervention with hydralazine to lower blood pressure in established hypertension did not readily reverse immune cell infiltration in the brain or cognitive impairment. However, it is plausible that a period of just 2 weeks of hydralazine administration is insufficient to resolve the brain inflammation that develops in response to angiotensin II-induced hypertension which may persist after the initial hypertensive stimulus has abated, consistent with several clinical effects of anti-hypertensive therapy on cognitive dysfunction (Stuhec et al., 2017). New strategies that target both blood pressure and inflammation may be a more effective approach to treat cognitive impairment.

It seems likely that breakdown of the blood-brain barrier was an important factor in hypertension-induced cognitive impairment. Blood-brain barrier dysfunction is associated with early cognitive decline in vascular cognitive impairment (Li et al., 2021) and Alzheimer's disease patients (Bowman et al., 2018) even in the absence of amyloid-β and tau changes (Nation et al., 2019). Our data reveal that in hypertensive mice the blood-brain barrier was compromised in the hippocampus and cortex, key brain regions involved in cognition. Besides facilitating immune cell infiltration, a leaky blood-brain barrier is also associated with pathological events such as white matter hyperintensities (Zhang et al., 2019) which can contribute to the development of cognitive impairment.

## 5 Conclusions

The present findings indicate that hypertensive stimuli can promote brain inflammation, cognitive impairment and changes to the brain transcriptome in a blood pressure-dependent manner. The processes involved in neuroinflammation during hypertension are complex and our findings suggest that blood-brain barrier breakdown and CCR2 expression are likely to be involved, and this was associated with the development of cognitive impairment. As we found no evidence that drug-induced attenuation of established hypertension could reduce brain inflammation or cognitive dysfunction, our study highlights the importance of managing hypertension to reduce the risk of developing neuroinflammation and cognitive impairment.

## CRediT authorship contribution statement

**Quynh Nhu Dinh:** Conceptualization, Formal analysis, Investigation, Methodology, Writing – original draft, Writing – review & editing. **Antony Vinh:** Formal analysis, Investigation, Methodology, Supervision, Writing – review & editing. **Cecilia Lo:** Formal analysis, Investigation, Methodology, Writing – review & editing. **David E. Wong Zhang:** Formal analysis, Investigation, Methodology, Writing – review & editing. **Hericka Bruna Figueiredo Galvao:** Formal analysis, Investigation, Writing – review & editing. **Sharmelee Selvaraji:** Formal analysis, Investigation, Writing – review & editing. **Hyun Ah Kim:** Formal analysis, Investigation, Methodology, Writing – review & editing. **Sophocles Chrissobolis:** Conceptualization, Formal analysis, Investigation, Supervision, Writing – review & editing. **Thiruma V. Arumugam:** Formal analysis, Investigation, Methodology, Supervision, Writing – review & editing. **Grant R. Drummond:** Conceptualization, Formal analysis, Supervision, Writing – review & editing. **Christopher G. Sobey:** Conceptualization, Formal analysis, Funding acquisition, Investigation, Supervision, Writing – review & editing. **T. Michael De Silva:** Conceptualization, Formal analysis, Investigation, Methodology, Supervision, Writing – original draft, Writing – review & editing.

## Sources of funding

This work was supported by Project Grants to 10.13039/100009772CGS from the 10.13039/501100000925National Health and Medical Research Council of Australia (NHMRC; GNT1064686 and GNT1085323) and a Senior Research Fellowship to 10.13039/100009772CGS (GNT1079467).

## Declaration of competing interest

The authors declare the following financial interests/personal relationships which may be considered as potential competing interests:Christopher G. Sobey reports financial support was provided by the 10.13039/501100000925National Health and Medical Research Council, Australia. If there are other authors, they declare that they have no known competing financial interests or personal relationships that could have appeared to influence the work reported in this paper.

## Data Availability

Data will be made available on request.
