# Supplementary material for: Hypertension promotes neuroinflammation, brain injury and cognitive impairment
Source: Brain Behav Immun Health. 2025 Jul 10;48:101059. doi: 10.1016/j.bbih.2025.101059 (PMC12281552; doi:10.1016/j.bbih.2025.101059)
Supplement: Multimedia component 1 [file mmc1.docx]

**SUPPLEMENTARY MATERIAL**

**HYPERTENSION PROMOTES BRAIN INFLAMMATION,
INJURY AND COGNITIVE IMPAIRMENT**

**Quynh Nhu Dinh^1,2^, Antony Vinh^1,2^, Cecilia Lo^2^, David E. Wong Zhang^1,2^, Hericka Bruna Figueiredo Galvao^1,2^, Sharmelee Selvaraji^3^, Hyun Ah Kim^1,2^, Sophocles Chrissobolis^4^, Thiruma V. Arumugam^1,2^, Grant R. Drummond^1,2^,
Christopher G. Sobey^1,2^, T. Michael De Silva^1,2^**

^1^Centre for Cardiovascular Biology and Disease Research and La Trobe Institute for Molecular Sciences (LIMS), La Trobe University, Victoria, Australia;

^2^Department of Microbiology, Anatomy, Physiology & Pharmacology, School of Agriculture, Biomedicine, Environment, La Trobe University, Victoria, Australia;

^3^Memory Aging and Cognition Centre, Department of Pharmacology, National University of Singapore, Singapore;

^4^Department of Pharmaceutical and Biomedical Sciences, Raabe College of Pharmacy, Ohio Northern University, Ohio, USA

Corresponding Author:

T. Michael De Silva, PhD

Dept. of Microbiology, Anatomy, Physiology &

Pharmacology
School of Agriculture, Biomedicine, Environment

La Trobe University

Bundoora, Victoria 3086, Australia

e-Mail: [t.desilva@latrobe.edu.au](mailto:t.desilva@latrobe.edu.au)

Phone: +61-3-94796876

Quynh Nhu Dinh: [q.dinh@latrobe.edu](mailto:q.dinh@latrobe.edu).au

Antony Vinh: [a.vinh@latrobe.edu.au](mailto:a.vinh@latrobe.edu.au)

Cecilia Lo: [c.desilva@latrobe.edu.au](mailto:c.desilva@latrobe.edu.au)

David E. Wong Zhang: [17141319@students.latrobe.edu.au](mailto:17141319@students.latrobe.edu.au)

Hericka Bruna Figueiredo Galvao [H.FigueiredoGalvao@latrobe.edu.au](mailto:H.FigueiredoGalvao@latrobe.edu.au)

Sharmelee Selvaraji: [e0358056@u.nus.edu](mailto:e0358056@u.nus.edu)

Hyun Ah Kim: [h.kim2@latrobe.edu.au](mailto:h.kim2@latrobe.edu.au)

Sophocles Chrissobolis: [s-chrissobolis@onu.edu](mailto:s-chrissobolis@onu.edu)

Thiruma V. Arumugam: [g.arumugam@latrobe.edu.au](mailto:g.arumugam@latrobe.edu.au)

Grant R. Drummond: [g.drummond@latrobe.edu.au](mailto:g.drummond@latrobe.edu.au)

Christopher G. Sobey: [c.sobey@latrobe.edu.au](mailto:c.sobey@latrobe.edu.au)

T. Michael De Silva: [t.desilva@latrobe.edu.au](mailto:t.desilva@latrobe.edu.au)


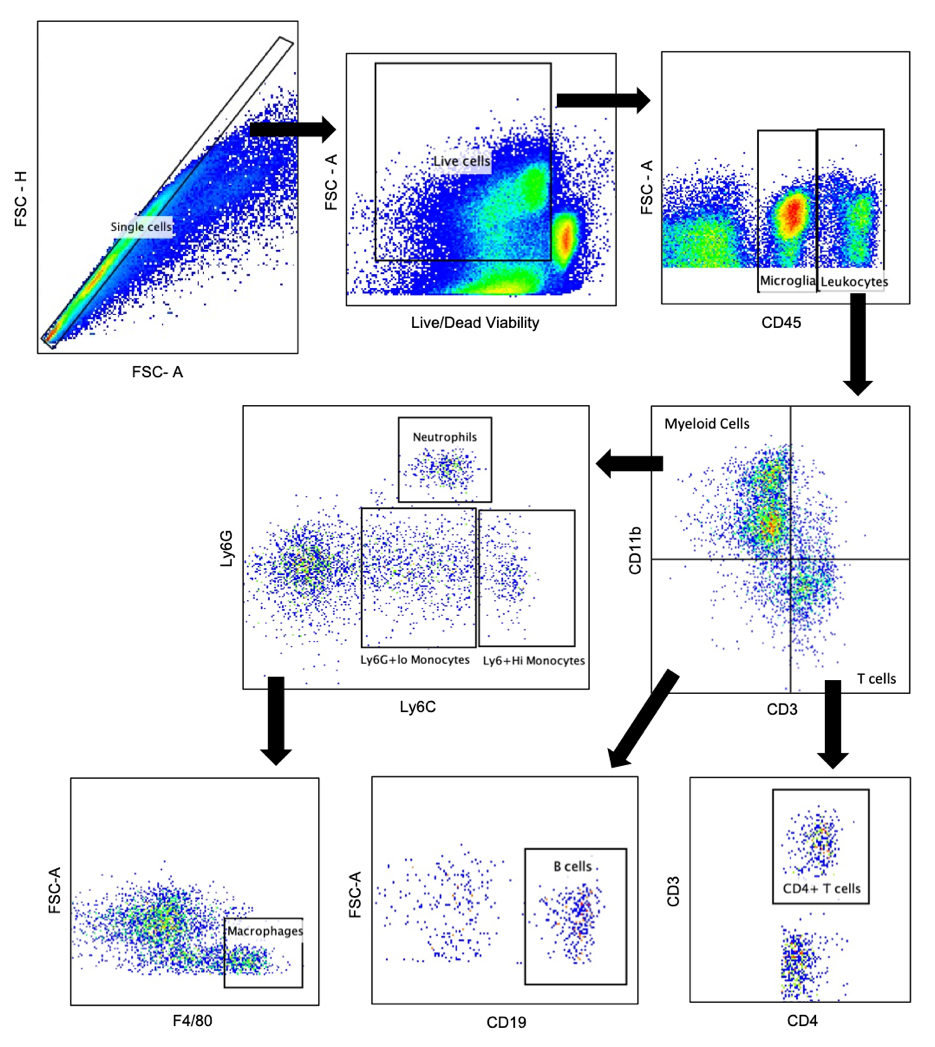


**Supplementary Figure 1. Gating strategy for flow cytometric analysis.** Single cells were gated by forward scatter (FSC)-height vs FSC-area and dead cells were excluded (live/dead stain). From this, leukocytes were gated as the CD45+high population and microglia were gated as the CD45+low population against FSC-area. Leukocytes were then further gated into myeloid cells (CD45+CD11b+), T cells (CD45+CD3+) and B cells (CD45+CD19+). The T cells were separated as T helper cells (CD45+CD3+CD4+). The myeloid cells were separated as neutrophils (CD45+CD11b+Ly6G+), monocytes (CD45+CD11b+Ly6C+) and macrophages (CD45+CD11b+F4/80+).


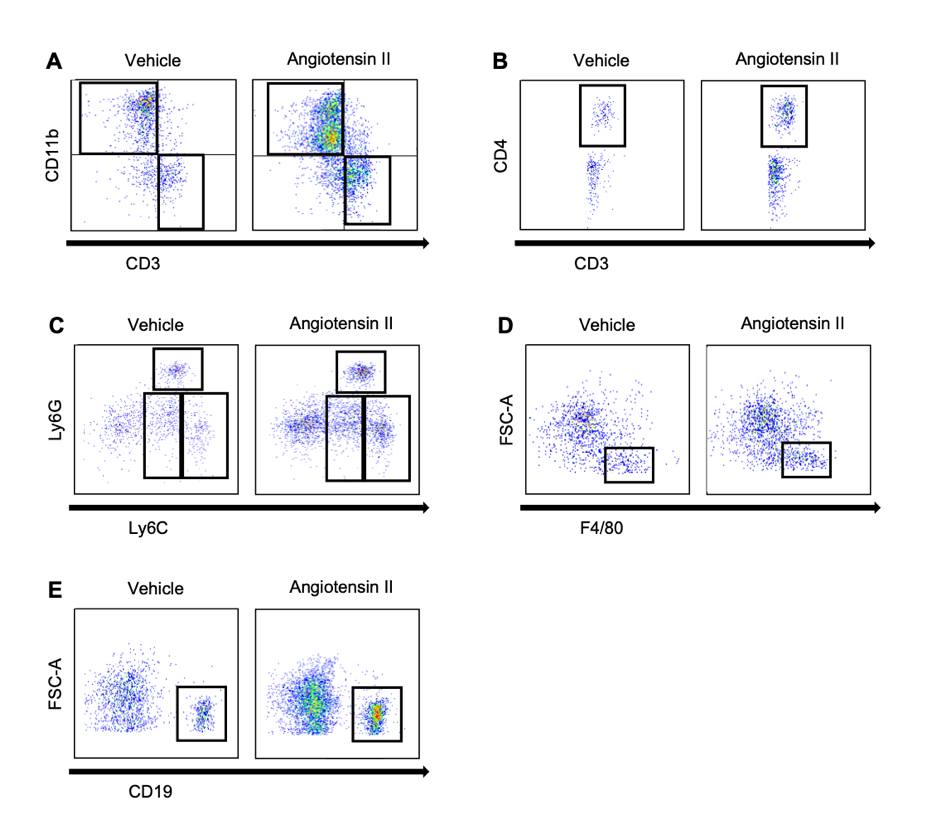


**Supplementary Figure 2. Angiotensin II infusion promotes immune cell infiltration in the brain.** Representative flow cytometry dot plots showing gating strategy for **A:** CD11b+ myeloid cells and CD3+ T cells, **B:** CD3+CD4+ T cells, **C:** Ly6G+ neutrophils and Ly6C+ monocytes, **D:** F4/80+ macrophages and **E:** CD19+ B cells in vehicle or angiotensin II infused mice.

**
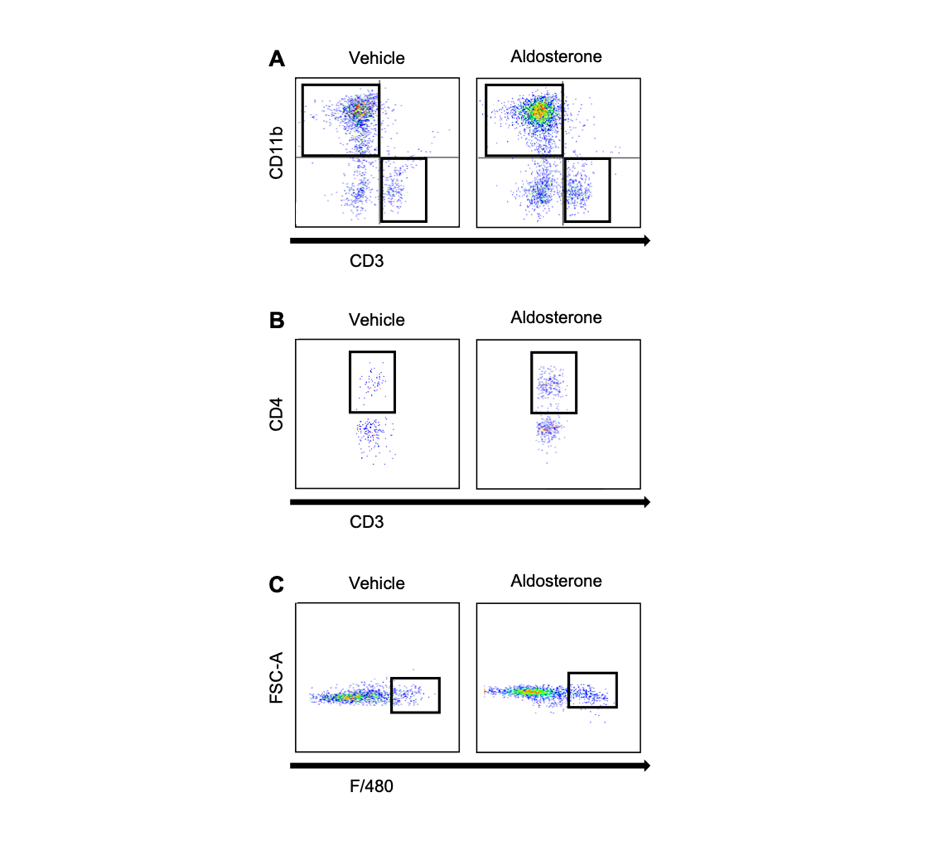
**

F4/80

**Supplementary Figure 3. Aldosterone II infusion promotes immune cell infiltration in the brain.** Representative flow cytometry dot plots showing gating strategy for **A:** CD11b+ myeloid cells and CD3+ T cells, **B:** CD3+CD4+ T cells and **C:** F4/80+ macrophages in vehicle or aldosterone infused mice.


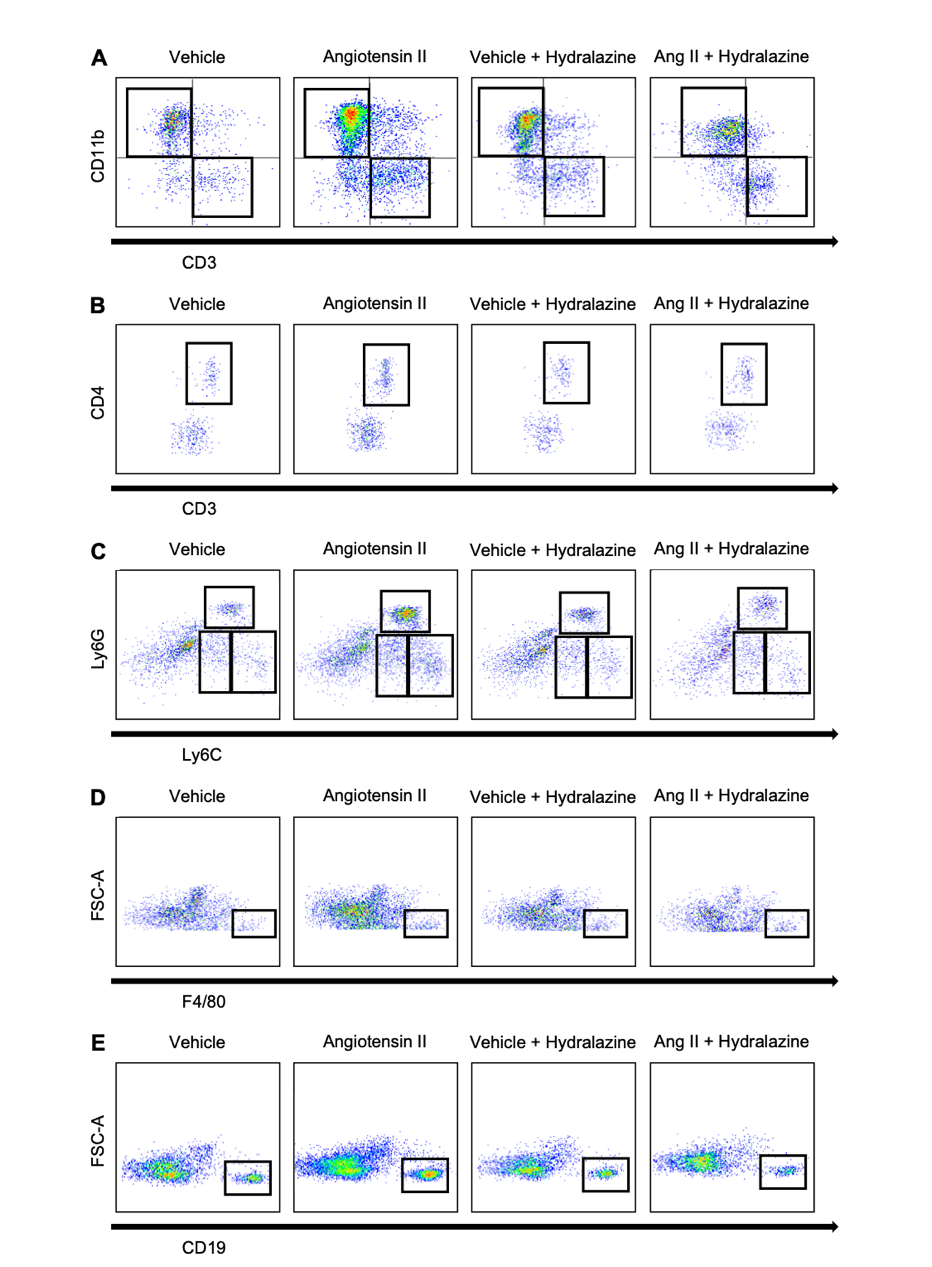


**Supplementary Figure 4. Angiotensin II-induced increase in brain immune cell infiltration is blood pressure-dependent.** Representative flow cytometry dot plots showing gating strategy for **A:** CD11b+ myeloid cells and CD3+ T cells, **B:** CD3+CD4+ T cells, **C:** Ly6G+ neutrophils and Ly6C+ monocytes, **D:** F4/80+ macrophages and **E:** CD19+ B cells in vehicle, angiotensin II, vehicle + hydralazine or angiotensin II + hydralazine infused mice.


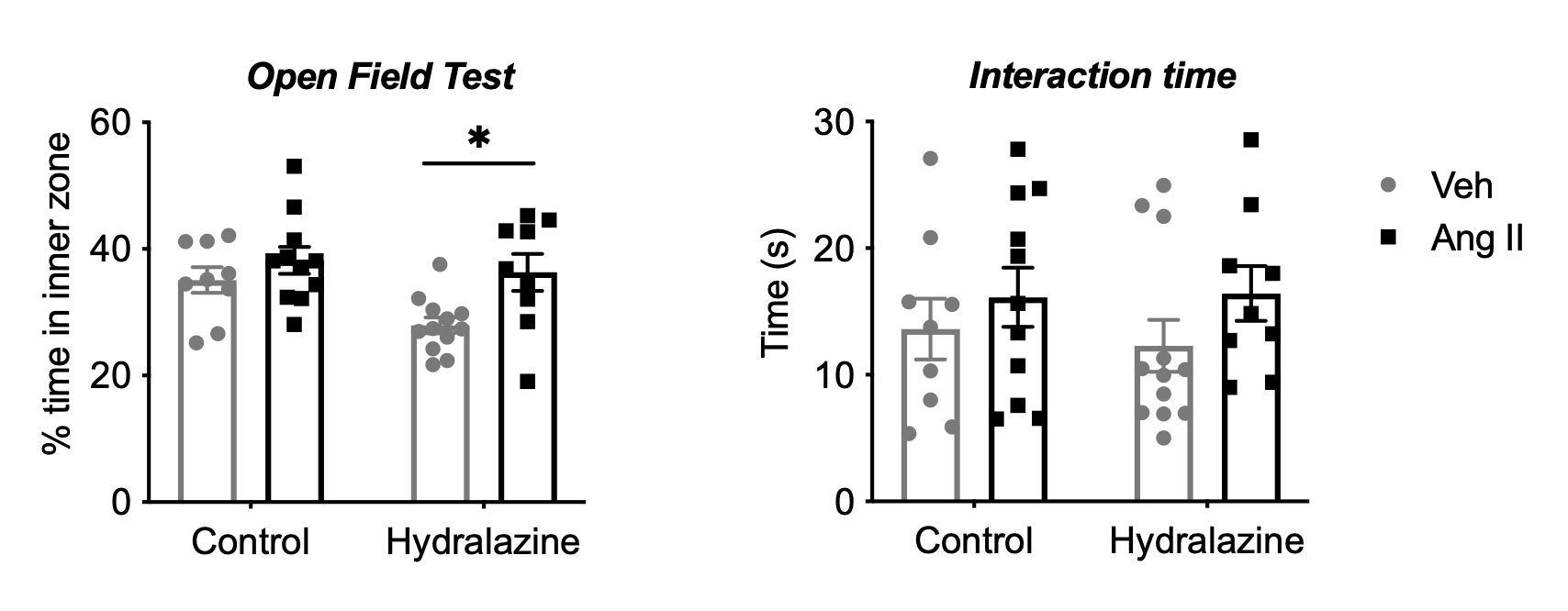


**B**

**A**

**Supplementary Figure 5.** **A:** percentage of time in the inner zone in the open field test (day 2 of habituation for the novel object recognition test) and **B:** total interaction time with both objects during the retention phase in vehicle, angiotensin II, vehicle + hydralazine or angiotensin II + hydralazine infused mice. **P<*0.05, n=9-12 per group, two-way ANOVA with a Sidak’s post-hoc test.

**B**

**A**


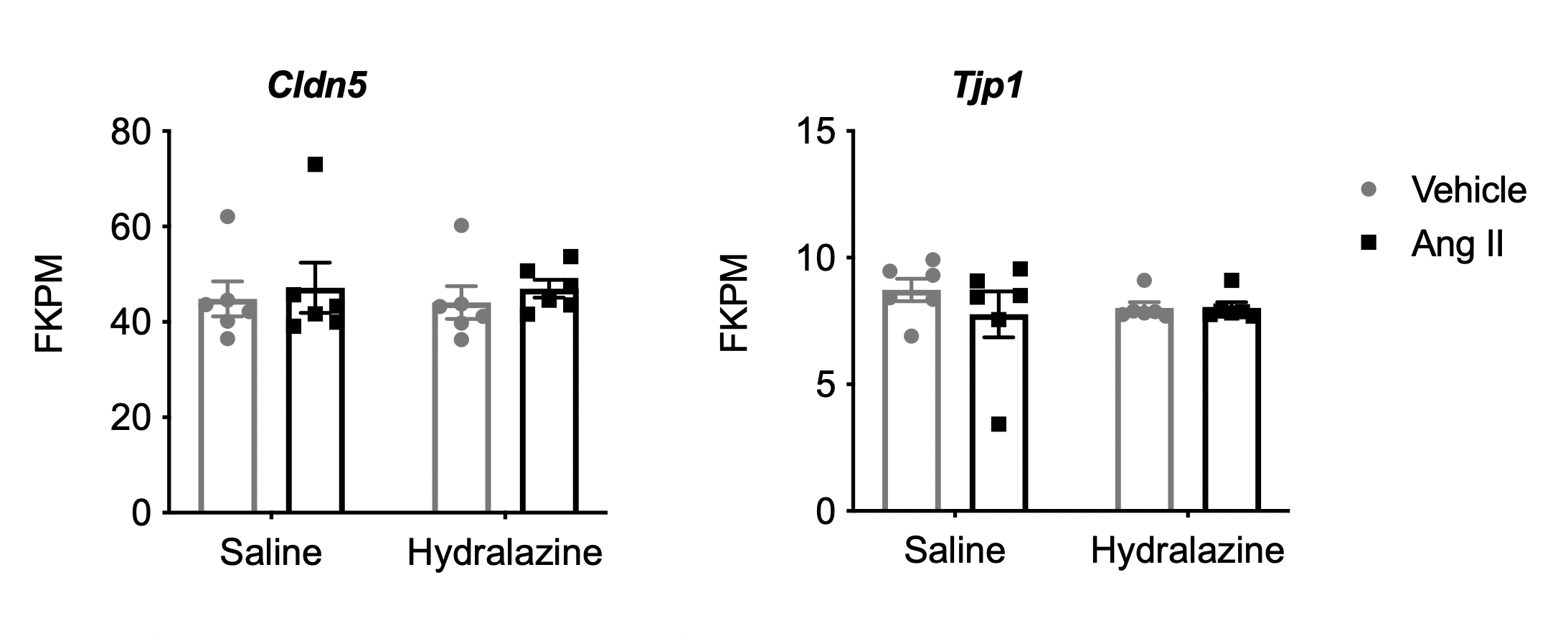


**Supplementary Figure 6.** Gene expression of **A:** *Cldn5* (claudin 5) and **B:** *Tjp1* (zonula occludens 1) in the brain of vehicle, angiotensin II, vehicle + hydralazine or angiotensin II + hydralazine infused mice. Data is presented as FKPM values from RNA sequencing analysis, n=6 per group.


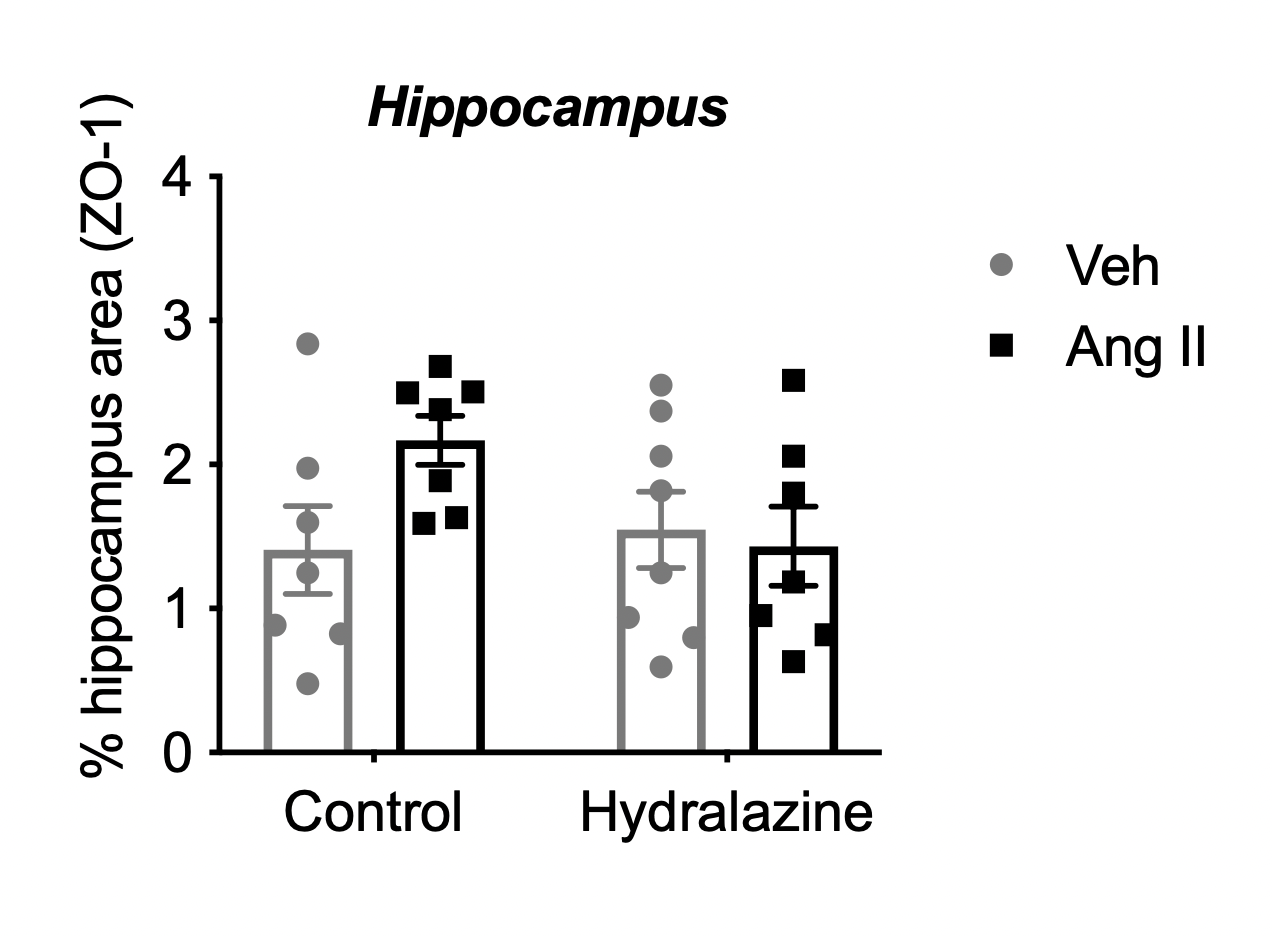


P=0.1


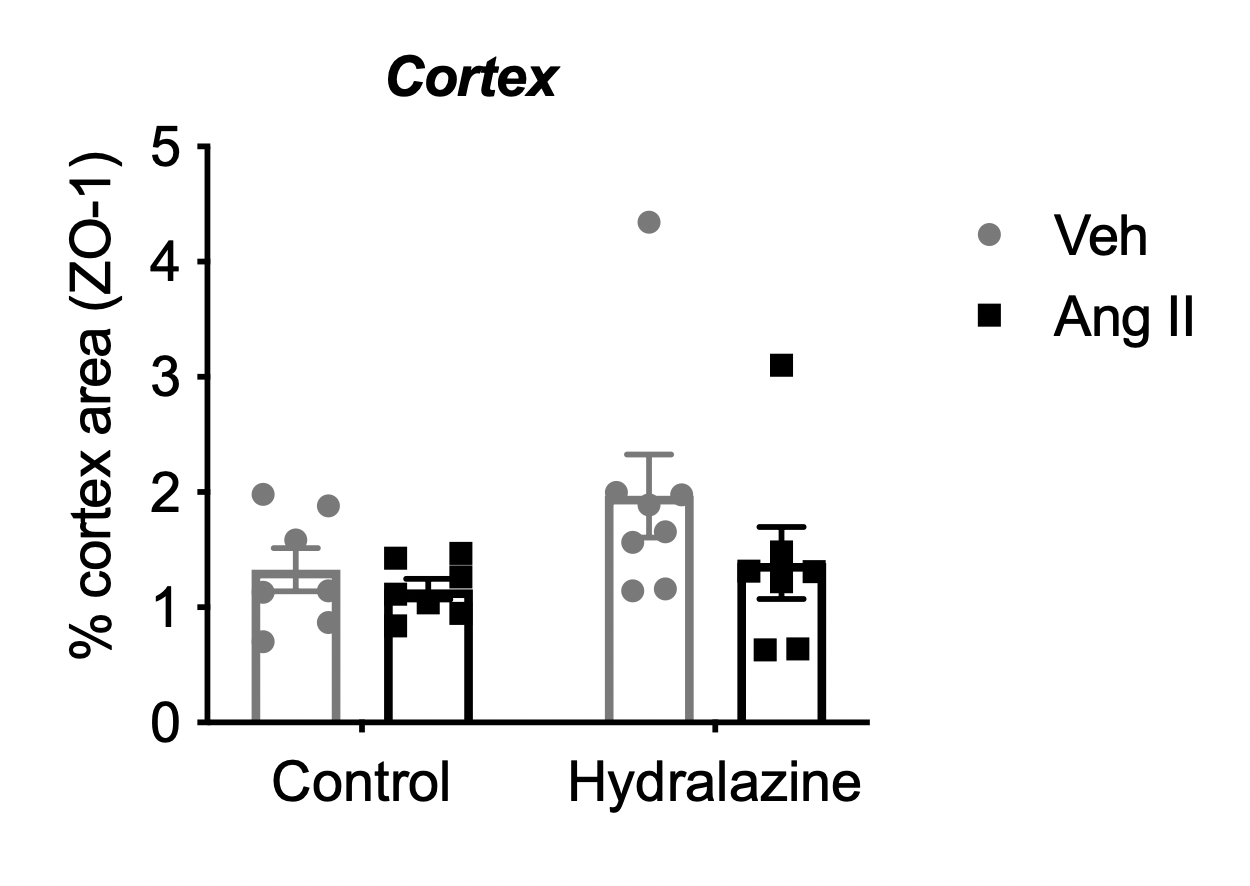


**Supplementary Figure 7.** Percentage-stained area of zonula occludens 1 (ZO-1) in the **A:** hippocampus and **B:** cortex of vehicle, angiotensin II, vehicle + hydralazine and angiotensin II + hydralazine. Representative images are shown above. Data is presented as % stained area. n=7-8 per group.

**
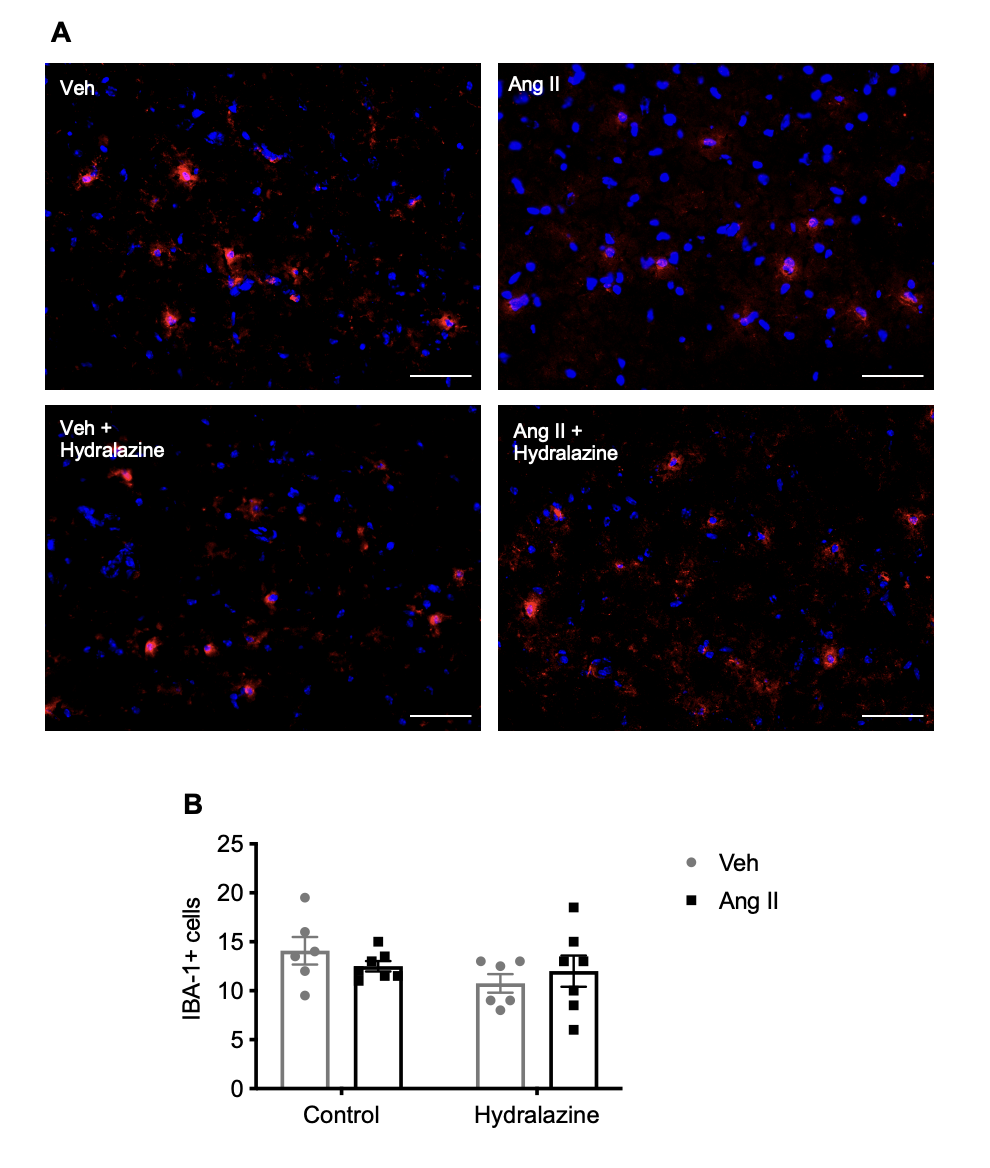
**

**Supplementary Figure 8.** **A:** Representative images and **B:** cell counts of Iba-1 staining in the CA1 region of the hippocampus of vehicle, angiotensin II, vehicle + hydralazine and angiotensin II + hydralazine mice (n=6-7). Data is mean ± S.E.M. Scale bar = 50 μm

**
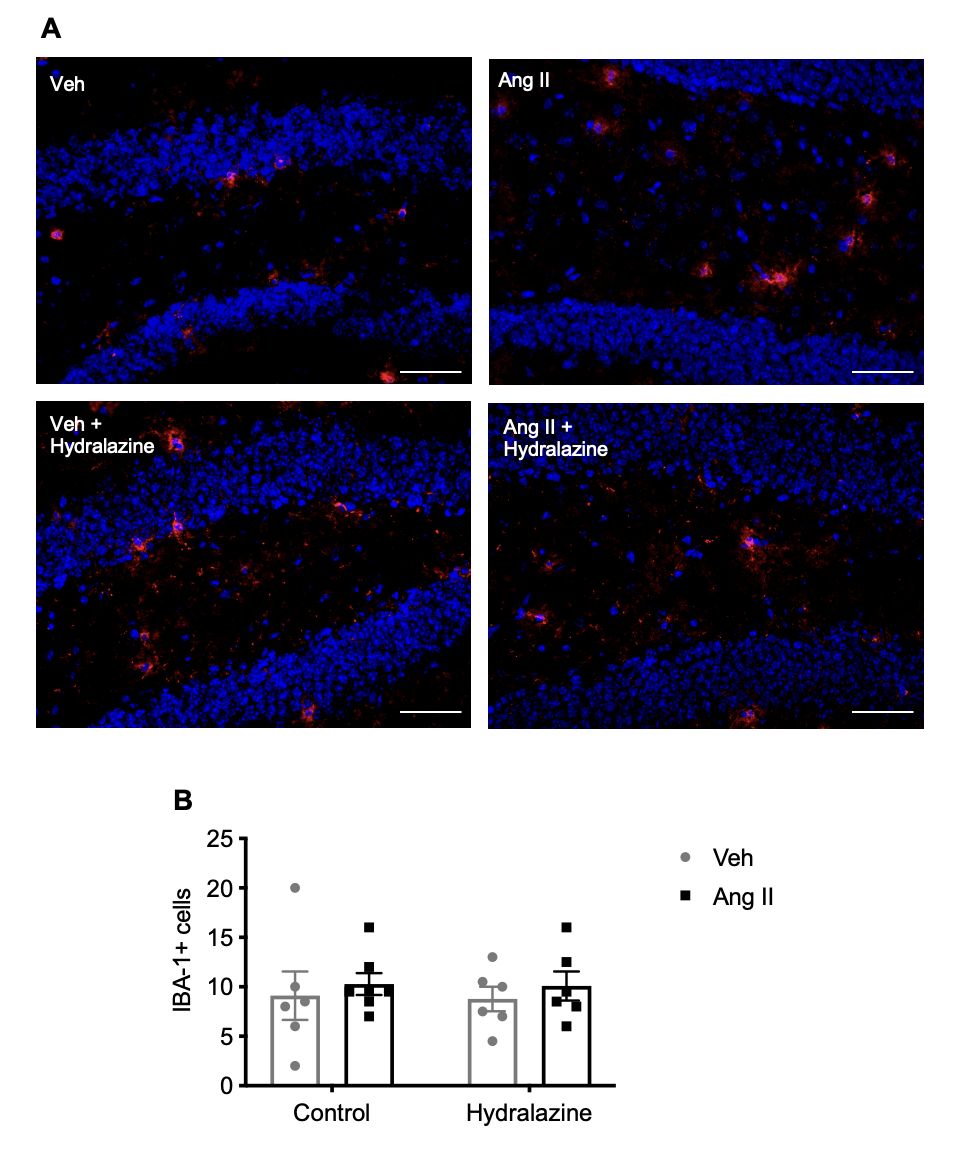
**

**Supplementary Figure 9.** **A:** Representative images and **B:** cell counts of Iba-1 staining in the dentate gyrus region of the hippocampus of vehicle, angiotensin II, vehicle + hydralazine and angiotensin II + hydralazine mice (n=6-7). Data is mean ± S.E.M. Scale bar = 50 μm


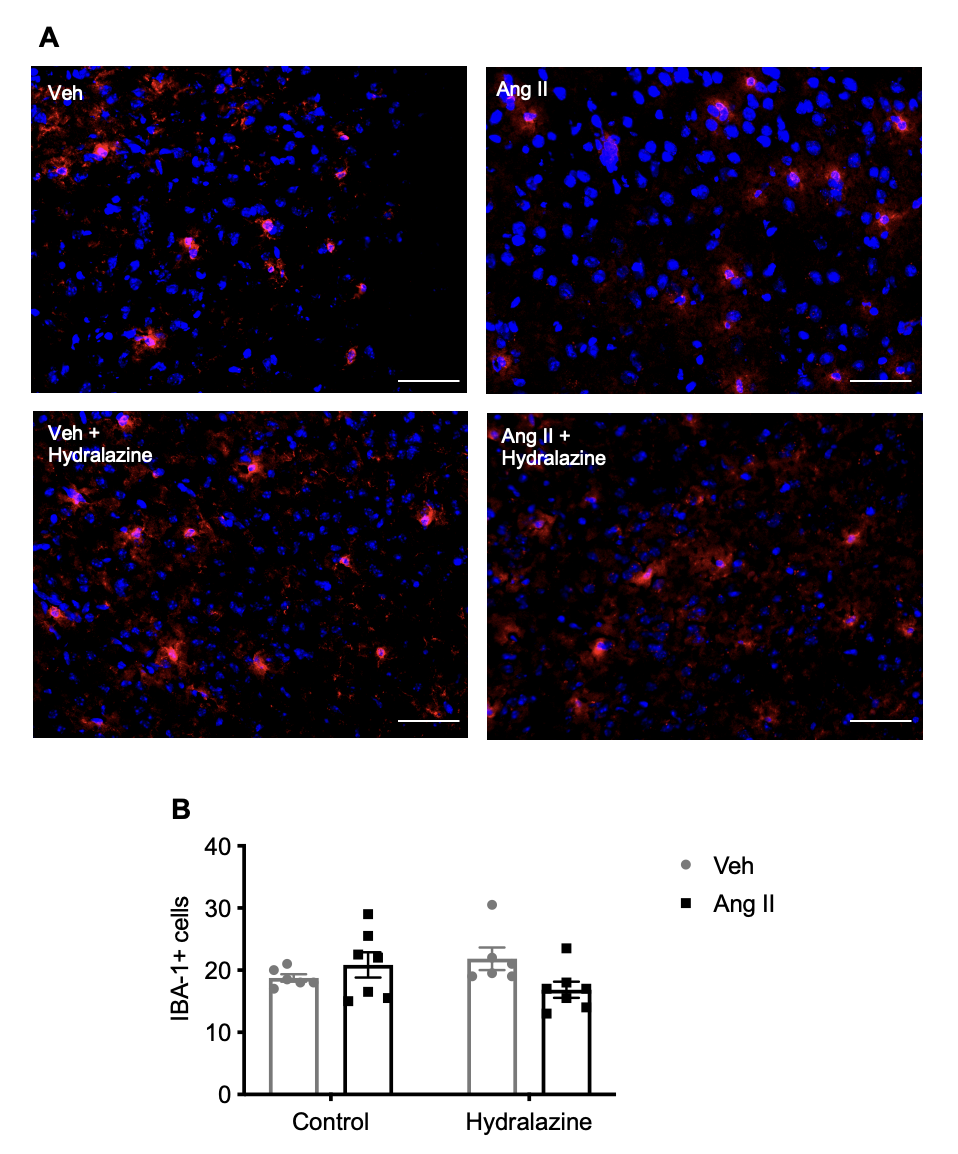


**Supplementary Figure 10.** **A:** Representative images and **B:** cell counts of Iba-1 staining in the cortex of vehicle, angiotensin II, vehicle + hydralazine and angiotensin II + hydralazine mice (n=6-7). Data is mean ± S.E.M. Scale bar = 50 μm

**
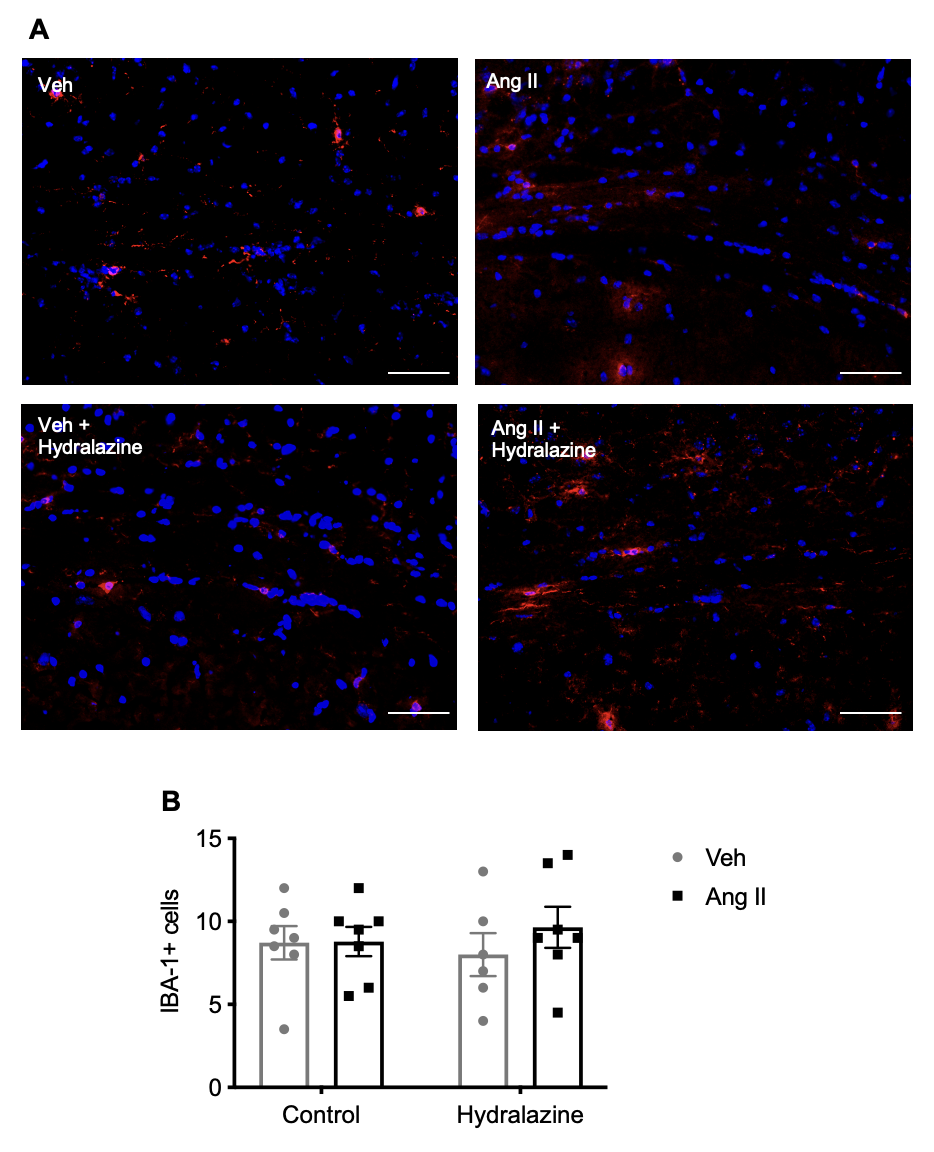
Supplementary Figure 11.** **A:** Representative images and **B:** cell counts of Iba-1 staining in the corpus callosum of vehicle, angiotensin II, vehicle + hydralazine and angiotensin II + hydralazine mice (n=6-7). Data is mean ± S.E.M. Scale bar = 50 μm


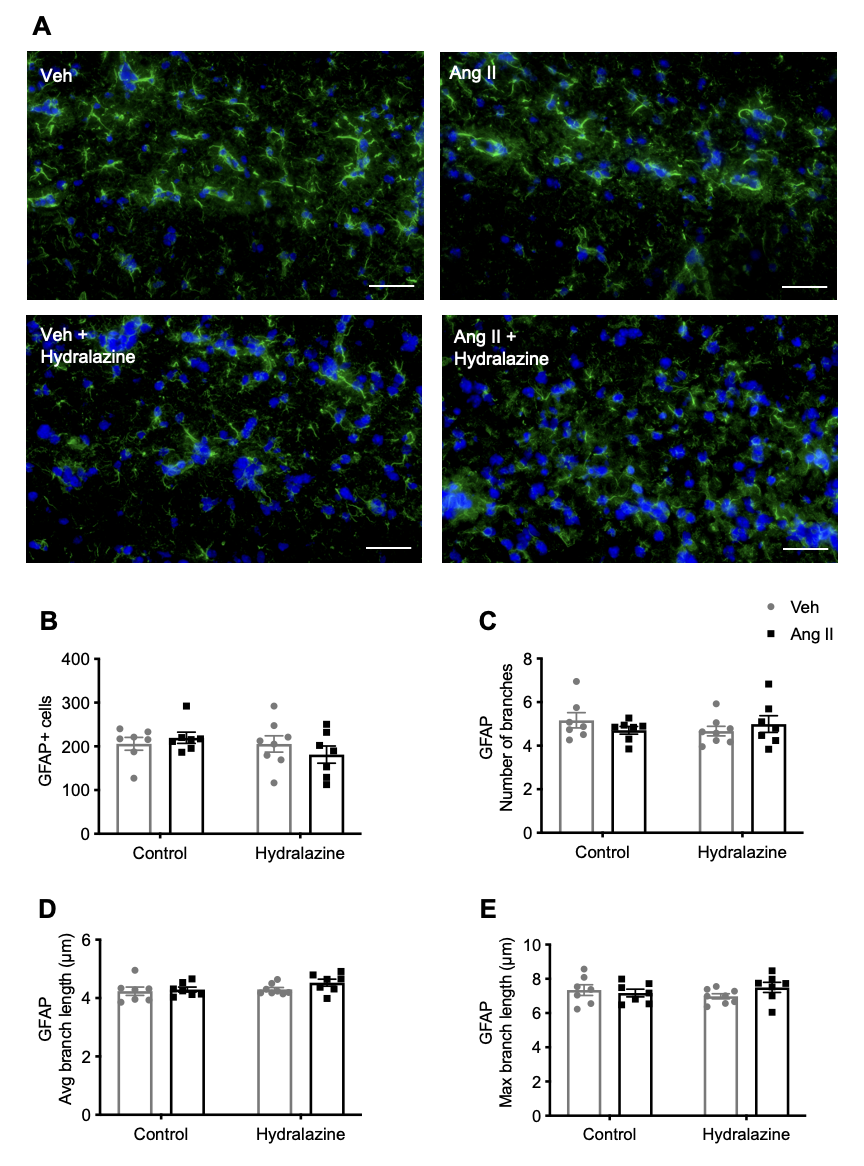


**Supplementary Figure 12.** **A:** Representative images, **B:** cell counts, **C:** number of branches, **D:** average branch length and **E:** max branch length of GFAP staining in the CA1 of the hippocampus of vehicle, angiotensin II, vehicle + hydralazine and angiotensin II + hydralazine mice (n=6-7). All data are mean ± S.E.M. Scale bar = 50 μm

**
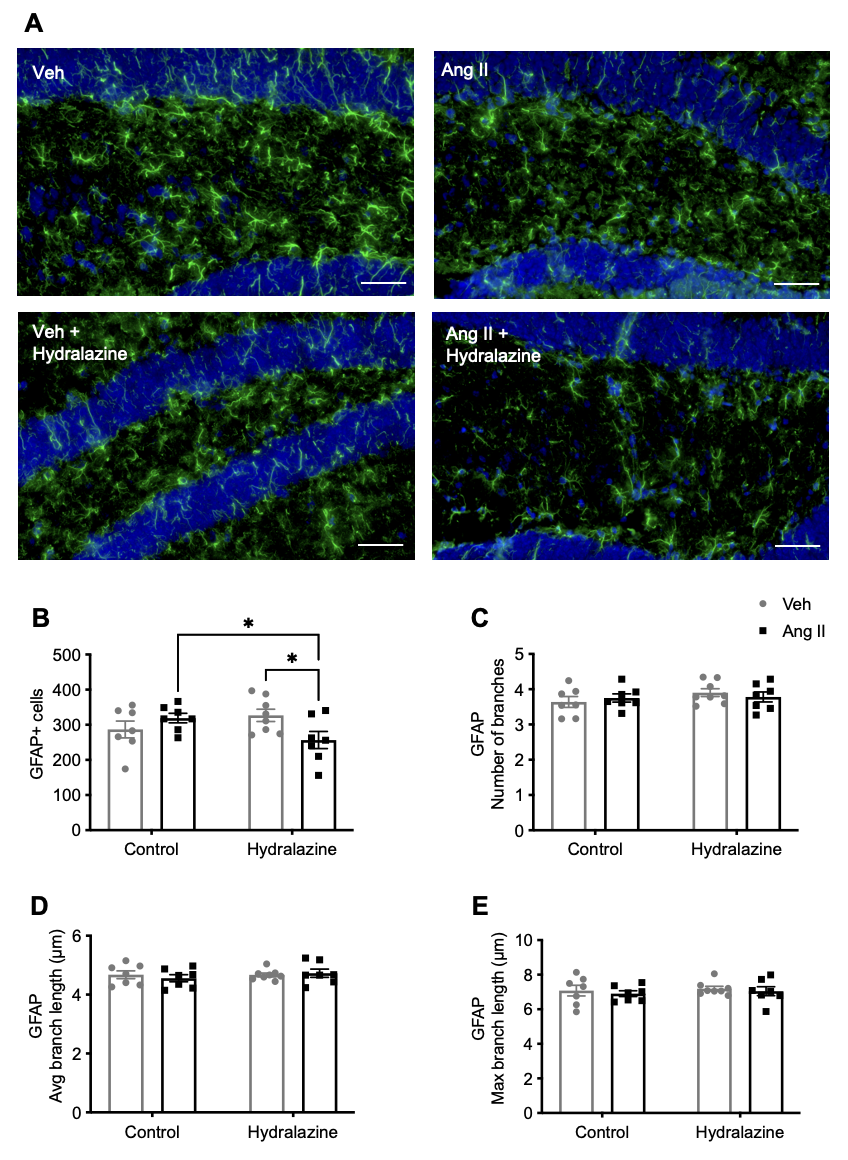
**

**Supplementary Figure 13.** **A:** Representative images, **B:** cell counts, **C:** number of branches, **D:** average branch length and **E:** max branch length of GFAP staining in the dentate gyrus of the hippocampus of vehicle, angiotensin II, vehicle + hydralazine and angiotensin II + hydralazine mice (n=6-7). All data are mean ± S.E.M. *P<0.05. Two-way ANOVA with Fisher’s LSD test. Scale bar = 50 μm

**
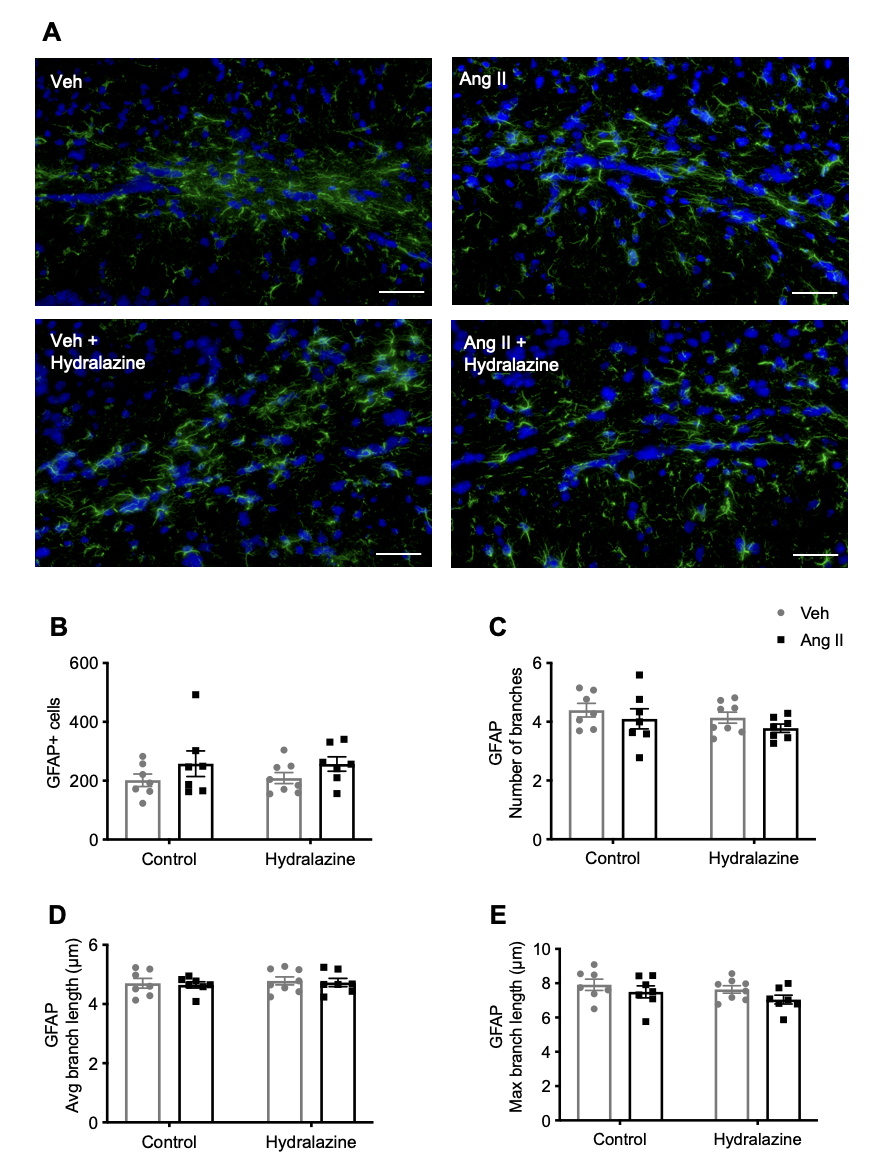
Supplementary Figure 14.** **A:** Representative images, **B:** cell counts, **C:** number of branches, **D:** average branch length and **E:** max branch length of GFAP staining in the corpus callosum of vehicle, angiotensin II, vehicle + hydralazine and angiotensin II + hydralazine mice (n=6-7). All data are mean ± S.E.M. Scale bar = 50 μm

**A**

***Angiotensin II vs vehicle – whole brain***

**
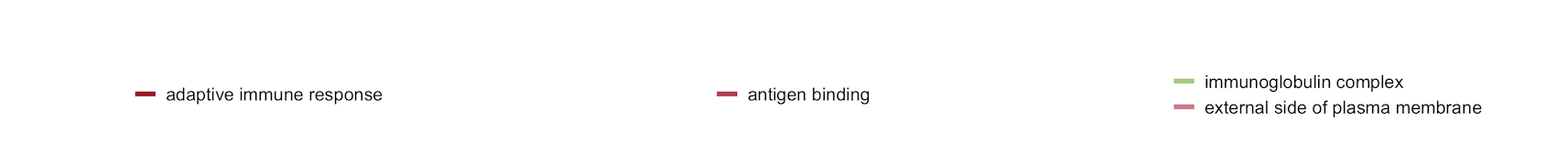

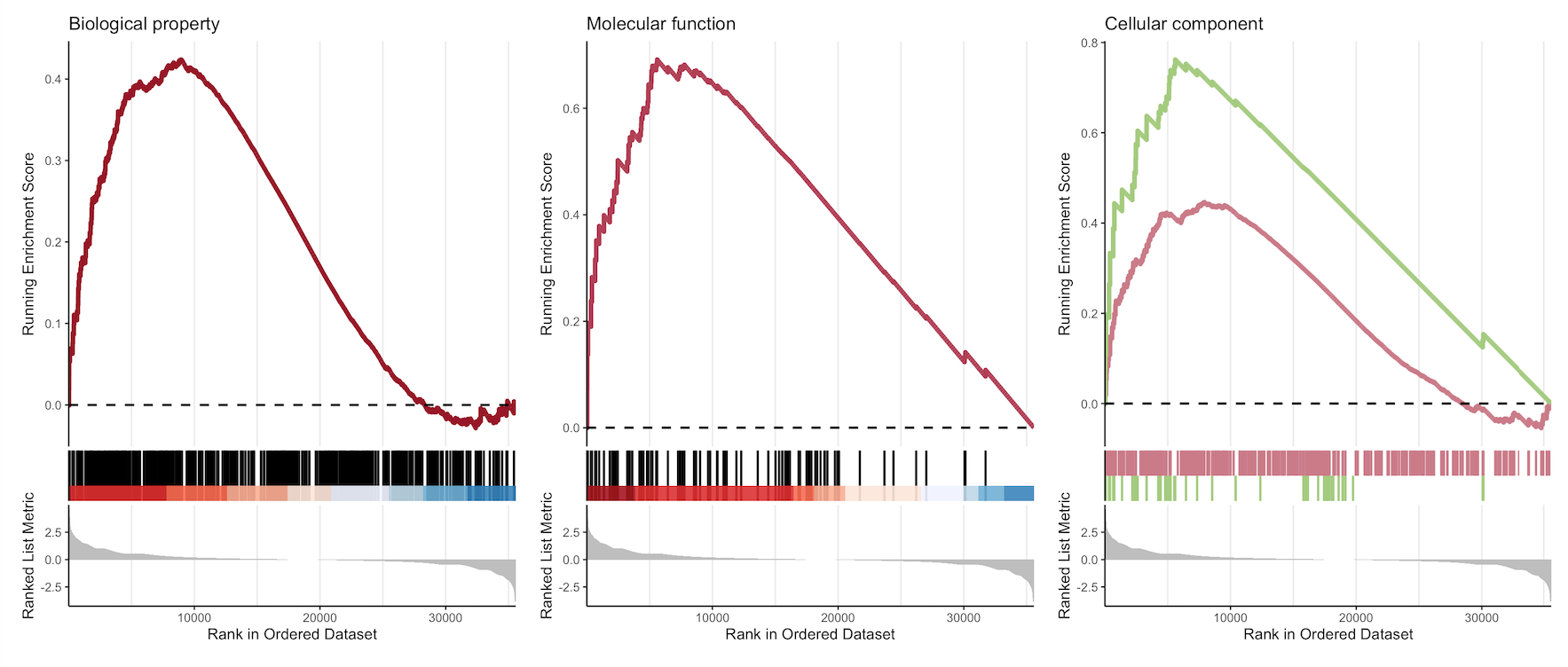
**

***Angiotensin II vs vehicle – hippocampus***

**B**

**
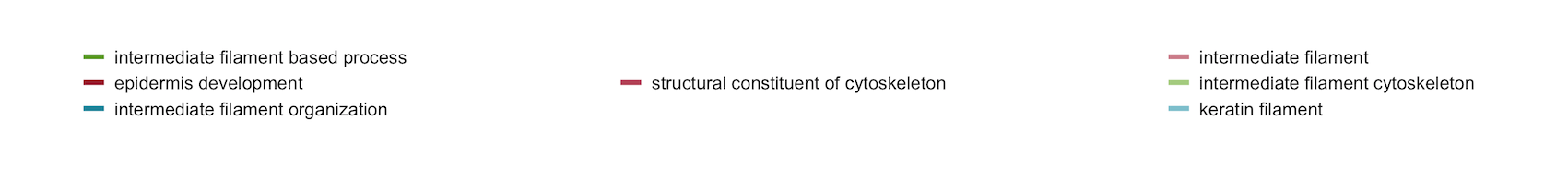

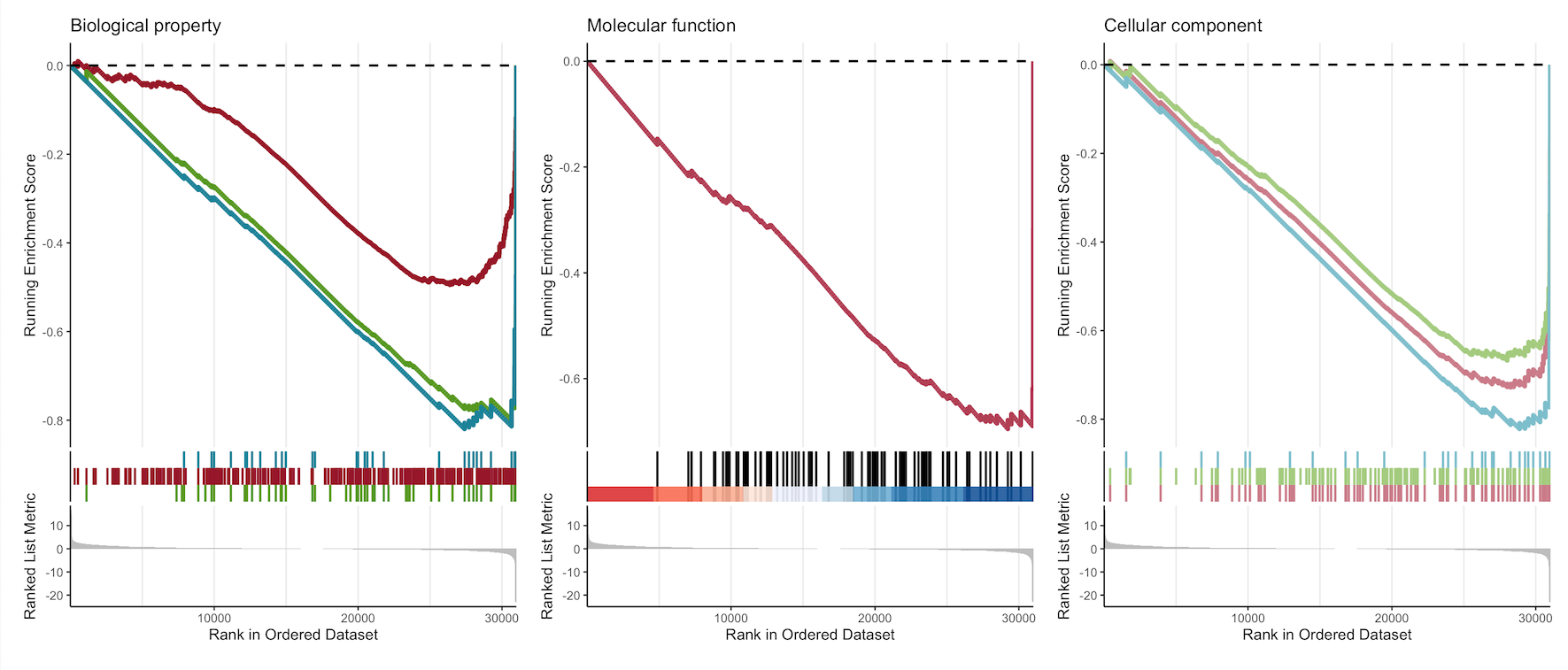
**

***Angiotensin II + hydralazine vs angiotensin II – hippocampus***

**C**

**
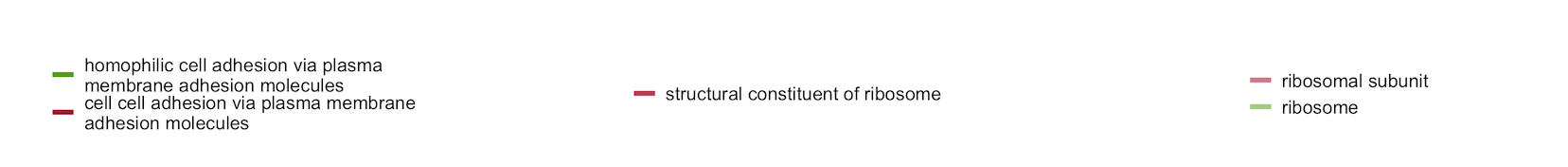

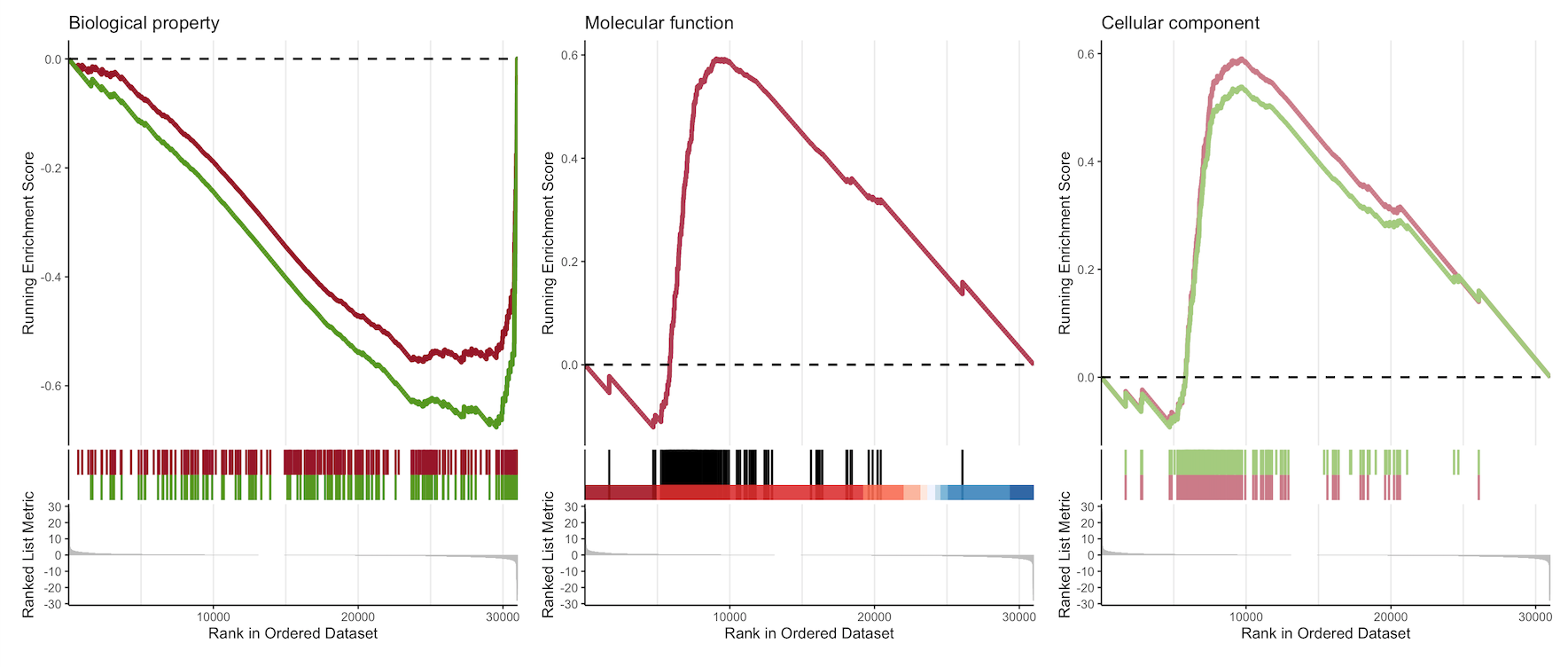
**

**Supplementary Figure 15.** GSEA comparison showing gene ontologies from **A:** the whole brain of angiotensin II vs vehicle treated mice, **B:** the hippocampus of angiotensin II vs vehicle treated mice, and **C:** the hippocampus of angiotensin II + hydralazine vs angiotensin II treated mice. Running enrichment scores are shown in the top plot of **A-C** where the leading edge displays whether a pathway is up- or down-regulated. Each vertical dash in the middle plot represents a gene expressed within a pathway and its location is determined by its rank from a gene list ordered by decreasing log_2_-fold values as shown in the bottom plot. Respective gene ontology terms are shown at the bottom of **A-C**.

**A**

**B**

**Supplementary Figure 16.** **A:** percentage of time in the inner zone in the open field test (day 2 of habituation for the novel object recognition test) and **B:** total interaction time with both objects during the retention phase in vehicle, angiotensin II, vehicle + hydralazine or angiotensin II + hydralazine infused mice. n=7-8 per group.
